# Leads in Arctic pack ice enable early phytoplankton blooms below snow-covered sea ice

**DOI:** 10.1038/srep40850

**Published:** 2017-01-19

**Authors:** Philipp Assmy, Mar Fernández-Méndez, Pedro Duarte, Amelie Meyer, Achim Randelhoff, Christopher J. Mundy, Lasse M. Olsen, Hanna M. Kauko, Allison Bailey, Melissa Chierici, Lana Cohen, Anthony P. Doulgeris, Jens K. Ehn, Agneta Fransson, Sebastian Gerland, Haakon Hop, Stephen R. Hudson, Nick Hughes, Polona Itkin, Geir Johnsen, Jennifer A. King, Boris P. Koch, Zoe Koenig, Slawomir Kwasniewski, Samuel R. Laney, Marcel Nicolaus, Alexey K. Pavlov, Christopher M. Polashenski, Christine Provost, Anja Rösel, Marthe Sandbu, Gunnar Spreen, Lars H. Smedsrud, Arild Sundfjord, Torbjørn Taskjelle, Agnieszka Tatarek, Jozef Wiktor, Penelope M. Wagner, Anette Wold, Harald Steen, Mats A. Granskog

**Affiliations:** 1 Norwegian Polar Institute, Fram Centre, 9296 Tromsø, Norway; 2 Department of Arctic and Marine Biology, Faculty of Biosciences, Fisheries and Economics, UiT The Arctic University of Norway, 9037 Tromsø, Norway; 3 Centre for Earth Observation Science, University of Manitoba, Winnipeg, MB R3T 2N2, Canada; 4 Institute of Marine Research, 9019 Tromsø, Norway; 5 Department of Physics and Technology, Faculty of Science and Technology, UiT The Arctic University of Norway, 9037 Tromsø, Norway; 6 Norwegian Meteorological Institute, 9239 Tromsø, Norway; 7 Centre for Autonomous Marine Operations and Systems, Department of Biology, Norwegian University of Science and Technology, 7491 Trondheim, Norway; 8 University Centre in Svalbard, Post box 156, 9171 Longyearbyen, Norway; 9 Alfred Wegener Institute, Helmholtz Center for Polar and Marine Research, 27570 Bremerhaven, Germany; 10 LOCEAN, UMR 7159, CNRS/UPMC/MNHN/IRD, Pierre and Marie Curie University, Paris cedex, France; 11 Institute of Oceanology, Polish Academy of Sciences, 81-712 Sopot, Poland; 12 Biology Department, Woods Hole Oceanographic Institution, Woods Hole, MA 02543, USA; 13 U.S. Army, Cold Regions Research and Engineering Laboratory, Hanover, NH 03755, USA; 14 Institute of Environmental Physics, University of Bremen, 28334 Bremen, Germany; 15 Bjerknes Centre for Climate Research, 5007 Bergen, Norway; 16 Geophysical Institute, University of Bergen, 5007 Bergen, Norway; 17 Department of Physics and Technology, University of Bergen, 5007 Bergen, Norway

## Abstract

The Arctic icescape is rapidly transforming from a thicker multiyear ice cover to a thinner and largely seasonal first-year ice cover with significant consequences for Arctic primary production. One critical challenge is to understand how productivity will change within the next decades. Recent studies have reported extensive phytoplankton blooms beneath ponded sea ice during summer, indicating that satellite-based Arctic annual primary production estimates may be significantly underestimated. Here we present a unique time-series of a phytoplankton spring bloom observed beneath snow-covered Arctic pack ice. The bloom, dominated by the haptophyte algae *Phaeocystis pouchetii*, caused near depletion of the surface nitrate inventory and a decline in dissolved inorganic carbon by 16 ± 6 g C m^−2^. Ocean circulation characteristics in the area indicated that the bloom developed *in situ* despite the snow-covered sea ice. Leads in the dynamic ice cover provided added sunlight necessary to initiate and sustain the bloom. Phytoplankton blooms beneath snow-covered ice might become more common and widespread in the future Arctic Ocean with frequent lead formation due to thinner and more dynamic sea ice despite projected increases in high-Arctic snowfall. This could alter productivity, marine food webs and carbon sequestration in the Arctic Ocean.

Annual phytoplankton net primary production in the Arctic Ocean has increased by 30% since the late 1990’s mainly due to the declining sea ice extent and an increasing phytoplankton growth season^1^. However, there is considerable uncertainty about the future change in Arctic Ocean primary productivity largely attributed to the different representation of the intricate balance between nutrient and light availability in coupled physical and biological ocean models^2^,^3^. The sea ice zone was identified as the area with largest model uncertainty^2^. Thus, a better understanding of the processes that control primary productivity in ice-covered waters will help to reduce this uncertainty.

Phytoplankton production beneath the ice-covered Arctic Ocean is assumed negligible because of the strong light attenuation properties of snow and sea ice, despite sporadic reports of phytoplankton growth beneath Arctic sea ice over the past decades^4^,^5^,^6^,^7^,^8^. This paradigm has recently been challenged by observations of under-ice phytoplankton blooms during the summer melt season^9^,^10^,^11^,^12^. In these studies, snowmelt onset and subsequent melt-pond formation permitted sufficient light transmission through the consolidated ice cover to trigger diatom-dominated phytoplankton blooms fuelled by underlying nutrient-rich waters^9^,^10^,^11^,^12^. In areas where extensive diatom blooms under thinning Arctic ice cover occur, current satellite-based estimates of annual primary production could be underestimated by an order of magnitude and change our perception of Arctic marine ecosystems^10^. In this study, we show for the first time that an under-ice phytoplankton bloom dominated by *Phaeocystis pouchetii* was actively growing beneath snow-covered pack ice at higher latitudes and earlier in the season than previously observed.

We studied the ice-associated ecosystem and the environmental factors shaping it in the Arctic Ocean north of Svalbard from 11 January to 24 June 2015 during the Norwegian young sea ICE (N-ICE2015) expedition^13^. Four ice camps were established during N-ICE2015^13^, but herein we focus on drifts of ice floes 3 and 4 covering early spring to early summer (Fig. 1a). Chlorophyll (Chl *a*) concentrations in the water column were low (<0.5 μg L^−1^) until 25 May when we first drifted into an under-ice phytoplankton bloom over the Yermak Plateau (YP) 80 km north of the ice edge (Fig. 1a) and remained within it until the end of the expedition on 22 June (Fig. 1b). The onset of the bloom coincided with shallowing of the pycnocline (Fig. 1b) and reduction in turbulent mixing (Table S1). This resulted in an increased residence time in the surface layer and thus light exposure of phytoplankton. Maximum Chl *a* concentrations of 7.5 μg L^−1^ were observed on 2 June and 50 m depth-integrated Chl *a* and particulate organic carbon (POC) standing stocks ranged between 109–233 mg Chl *a* m^−2^ and 9–22 g C m^−2^. The under-ice bloom (10–80 km from open waters) nearly depleted the surface nitrate inventory (Fig. 1c) and reduced dissolved inorganic carbon (DIC) at depths down to 50 m (Fig. S1). The depth of nutrient depletion clearly indicates drawdown by phytoplankton rather than ice algal growth. Indeed, the ice algal community, dominated by pennate diatoms, was distinct from the under-ice bloom. The under-ice bloom was dominated by *P. pouchetii* (Fig. 2a), which accounted for 55–92% of phytoplankton abundance and 12–93% of phytoplankton biomass and occurred both in its flagellate stage (Fig. 2b) and as large colonies (Fig. 2c). Furthermore, ice algal standing stocks were low (<3 mg Chl *a* m^−2^) throughout the drift indicating that contributions from the ice to water column stocks were negligible. A detailed list of protist plankton taxa observed during the bloom period can be found in the Supplementary Information (Table S2).

Regional ice thickness surveys with radius up to 50 km from the ice camp showed a total (ice plus snow) modal thickness of 1.8 m, with a secondary mode at 0.2 m, representing thin, lead ice (Fig. S2). Local surveys on floes 3 and 4 agreed, showing a modal ice thickness of 1.46 ± 0.66 m for the thick ice, covered by 0.39 ± 0.21 m of snow (Fig. S2), while snow thickness on the thin ice ranged from 0.01–0.06 m. Thus, for modelling of the under-ice light field and primary production, we treat all ice as being one of these two modal types either ‘thick ice’ with thick snow cover or ‘thin ice’ representative of recently refrozen leads with thin snow cover. The dominant snow-covered thick ice transmitted, on average, only <1% of the incident photosynthetic active radiation (E_PAR_) to the underlying water column. On the other hand, E_PAR_ transmittance for thin ice examined near camp in a refrozen lead was 20% on average, ranging from 6.3–42.2%. Leads in the ice pack (Fig. S3) were frequently created by ice divergence events (Fig. S5) prior to and during the bloom period. This high lead fraction is characteristic of the pack ice north of Svalbard^14^. Satellite-based ice type classification (Fig. S4 and Table S3) indicated that open water and thin, newly formed ice covered 1–33% of the area during the bloom period (Fig. 3a). Melt ponds were not a major factor in light availability during this study. Snowmelt did not start until early June and melt ponds formed only towards the end of the study period, covering <10% of the ice surface. We combined the estimated aerial fractions of open water, thin ice and thick, snow-covered ice with E_PAR_ transmittance through these surfaces to estimate the aggregate light field (Fig. 3a and Figs S6 and S7) experienced by phytoplankton.

The growth potential of *P. pouchetii* was modelled based on ^14^C photosynthesis-irradiance (PE) relationships obtained from a *P. pouchetii* bloom in the Greenland Sea^15^, taking into account the underwater light field based on measured^16^ and modelled irradiance through three different surface types (open water, thin ice with thin snow cover and thicker ice with thick snow cover) encountered during the study. The primary production (PP) model supports the observation that the bloom was actively growing beneath the ice despite the low irradiance (Fig. 3b). This is in accordance with previous studies showing that *Phaeocystis* is particularly well adapted to low light environments^17^,^18^. *In vivo* photosynthetic parameters, obtained with the Pulse Amplitude Modulation (PAM) method to assess the photo-acclimation status of the bloom, corroborate this finding (Table S5). High maximum quantum yields of charge separation in photosystem II (Chl *a* fluorescence of dark-acclimated cells) of 0.48–0.66 showed that the bloom-forming species were in good condition and actively growing. The maximum light utilization coefficient (α) of 0.188–0.295, obtained from Rapid Light Curves, also illustrates that the bloom exhibited high photosynthetic rates at low irradiances. Furthermore, the low POC/Chl *a* ratio of 31.4 in the upper 25 m of the under-ice water column suggests a relatively high investment in photosynthetic pigments, indicative of shade-acclimation. On the other hand, light saturation (E_k_) values of 137–584 μmol photons m^−2^ s^−1^ suggests that the phytoplankton community was at the same time acclimated to relatively high irradiances. This apparent inconsistency can be explained by the plasticity in photosynthetic performance of *P. pouchetii* that seems to be a characteristic feature of this species^15^ promoting its dominance under the highly variable light regime encountered during this study. The relatively minor contribution of diatoms to the under-ice bloom (Fig. 2a), with the exception of 8 June, is supported by the PP model results (Table S4). Diatoms are usually a major component of the phytoplankton spring bloom in the marginal ice zone north of Svalbard^19^ and have been reported to dominate under-ice blooms below ponded ice in summer^9^,^10^,^11^,^12^. The dynamic light conditions beneath the snow-covered drifting pack ice interspersed with transparent leads were apparently not sufficient to sustain growth rates for diatom bloom build-up^20^. Silicic acid concentrations in the upper 50 m during the bloom period remained close to winter values at 4.0 ± 0.4 μmol L^−1^ (Fig. S8), suggesting that no substantial diatom growth had taken place in these waters.

Measurements made with a vessel-mounted profiling current meter during the drifts over the YP indicated that transport velocities in Polar Surface Water (PSW) were weak. Time-mean current velocity components in PSW at 20–30 m depth for the bloom period were 2.2 cm s^−1^ heading nearly due west (Table S6). While these observations do not explicitly cover areas upstream of the drift itself, they indicate that advection over this part of YP was very weak during the expedition. An operational ocean model (PSY4, Mercator-Ocean, Table S6) shows similar, but smaller, net currents due west (Fig. 3c) at the same depth. These simulations do not contain tidal forcing and thus no tidal residual currents.

Model and observations both suggest that surface waters over the interior YP were not advected from open water regions. During the bloom, model and observations show the presence of Atlantic Water (AW) masses at greater depths (Fig. 3d). The overall circulation regime was not favourable for rapid advection of AW from the main branches of the West Spitsbergen Current into the interior part of the YP. Mean currents on the YP itself were weak and not capable of advecting substantial volumes of surface waters from the ice edge to the northernmost part of the observed bloom on time scales less than six weeks. Six weeks prior to the observed under-ice bloom (12 and 13 April), we measured Chl *a* concentrations of <0.1 μg L^−1^ in open waters across the shelf slope north of Svalbard on transit to floe 3. Thus, the weak re-circulation pattern over YP implies that the bloom grew *in situ* beneath the ice pack. The area that floes 3 and 4 drifted over towards the end of their respective drifts in June was open water in mid-April when the ice edge was at its northern-most position during the period April to June 2015 (Fig. 1a and Supplementary Video). Considering the low Chl *a* concentration measured in April, our observations also discount the alternative explanation that the bloom developed in open waters and was subsequently covered by drifting sea ice. However, enhanced vertical mixing over the YP^21^ supports the theory that *P. pouchetii* cells were likely mixed upwards from the sub-surface AW into the bloom in the PSW, thus contributing to the seeding of the bloom. This is consistent with observations that *P. pouchetii* is affiliated with AW^22^. Furthermore, in winter AW can be found close to the surface over the southern parts of YP providing another potential seeding mechanism.

The mean integrated drawdown of 16 ± 6 g C m^−2^ in the DIC inventory and a nitrate uptake equivalent to 15 ± 5 g C m^−2^ for the bloom period agreed well with the build-up in POC standing stocks. The biogeochemical footprint of the bloom was comparable to a diatom bloom beneath ponded, more transparent sea ice at a lower latitude^12^. Carbon export rates at 100 m increased from 74 to 244 mg C m^−2^ d^−1^ during the bloom period. Inspection of 100 m depth sediment-trap material revealed that the bulk of vertical carbon export was mediated via *P. pouchetii* aggregates, while zooplankton faecal pellets accounted for <2%. Out of the 63 zooplankton taxa identified in 200 μm MultiNet samples taken during the bloom period, the three dominant *Calanus* species (*C. finmarchicus, C. glacialis* and *C. hyperboreus*) accounted for 89 ± 8% of the total zooplankton biomass. The apparently low grazing impact on the *P. pouchetii* bloom by the dominant *Calanus* copepods is supported by the finding that *P. pouchetii* does not significantly contribute to *Calanus* diet^23^. Daily export rates were low, corresponding to 0.9–2% of POC standing stocks in the upper 100 m. This is consistent with previous measurements from the Barents Sea^24^ and supports the finding that *P. pouchetii* does not contribute much to deep carbon sequestration, which is generally mediated by diatoms^25^,^26^. This is corroborated by the dominance of fatty acid trophic markers from diatoms, rather than *Phaeocystis*, in benthic macrofauna^27^. Significant export of *P. pouchetii* biomass below 100 m has been reported previously^28^,^29^, but has been attributed to downwelling^29^, deep vertical mixing^26^ or could potentially be attributed to other mechanisms facilitating deep export such as mineral ballasting.

Our observations extend the spatial and temporal domains of known under-ice blooms. High lead fraction provided sufficient light to initiate and sustain an under-ice spring bloom dominated by *P. pouchetii,* despite the thick snow cover and limited light transmission. High lead fractions in Fram Strait, the Barents Sea, and in other parts of the Arctic Ocean^14^ suggest that early phytoplankton blooms under snow-covered sea ice might be widespread and become more prevalent in the future Arctic Ocean under an increasingly thinner and dynamic ice cover^30^ and a projected increase in high-Arctic snowfall^31^. This trend could be reinforced by the recent increase in advective transport of AW into the European Arctic^32^ seeding PSW with shade-adapted *P. pouchetii*, a conjecture that is corroborated by a shift in dominance from diatoms towards *P. pouchetii* in Fram Strait since 2006^33^,^34^,^35^. Nutrient depletion by early *P. pouchetii* blooms under snow-covered sea ice would constrain diatom blooms during the melt season, with far-reaching repercussions on bloom timing and composition, strength of the biological carbon pump and energy flow through Arctic marine food webs.

## Methods

### Standard analytical procedures

Chl *a* samples were collected on 25-mm GF/F filters (Whatman), extracted in 100% methanol for 12 h at 5 °C on board the ship and measured fluorometrically with an AU10 Turner Fluorometer (Turner Design, Inc.). Phaeopigments were measured by fluorescence after acidification with 5% HCl. Calibration of the Turner Fluorometer was carried out following the JGOFS protocol^36^. Chl *a* measurement uncertainty (5.5% of measured values) was estimated from triplicate water samples taken from depths ranging between 5 and 100 m. Particulate organic carbon (POC) and particulate organic nitrogen (PON) samples were collected onto pre-combusted 25-mm GF/F filters (Whatman), dried at 60 °C and stored at room temperature in PALL filter slides until analysis with continuous-flow mass spectrometry (CF-IMRS) carried out with a Roboprep/tracermass mass spectrometer (Europa Scientific, UK). All POC/N values were corrected for instrument drift and blanks. Water samples for inorganic nutrients were collected in 20 mL scintillation vials, fixed with 0.2 mL chloroform and stored refrigerated until sample analysis approximately 6 months later. Nitrite, nitrate, phosphate and silicic acid were measured spectrophotometrically at 540, 540, 810 and 810 nm, respectively, on a modified Scalar autoanalyser. The measurement uncertainty for nitrite is 0.06 μmol L^−1^ and 10% or less for nitrate, phosphate and silicic acid. Seawater for DIC analyses was sampled in 250 mL borosilicate bottles, preserved with 60 μL saturated mercuric chloride solution and stored dark and cool. DIC was determined using gas extraction followed by colourometric detection^37^. Certified Reference Material (CRM from A. Dickson at Scripps Institution of Oceanography, USA) was used for calibration and to check the accuracy of the analysis. The integrated nutrient drawdown in the upper 50 m for the bloom period was estimated from salinity-normalized (34.33) nDIC and 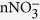 (nitrate) for all stations and converted to carbon using the measured POC/PON ratio of 5.7 ± 1.3. The complete N-ICE2015 water column biogeochemical dataset has been published in the Norwegian Polar Data Centre^38^.

### Sediment traps

Ice-tethered sediment traps (KC Denmark) were deployed four times at 5, 25, 50 and 100 m depth during the bloom period. Deployment time varied between 36 and 72 h, but was usually close to 48 h. Before deployment, each trap cylinder was filled with a saturated NaCl solution to reduce microbial activity and thus increase the retention of organic matter. The traps were carefully deployed and retrieved to avoid loss of trap material. Swimmers (copepods and other zooplankton) were removed before sub-sampling for Chl *a*, POC, plankton taxonomy, and faecal pellets.

### Phyto-PAM measurements

The maximum quantum yield of charge separation in photosystem II Chl *a* fluorescence (ΦPSII-max), the light saturation parameter (E_k_), the maximum light utilization coefficient (α) and the maximum relative electron transfer rate (rETR_max_) were obtained using the Pulse Amplitude Modulated (PAM) fluorometry method with a Phyto-PAM (Walz, Germany) following established protocols^39^.

### Phyto- and zooplankton analysis

**Figure 2:**
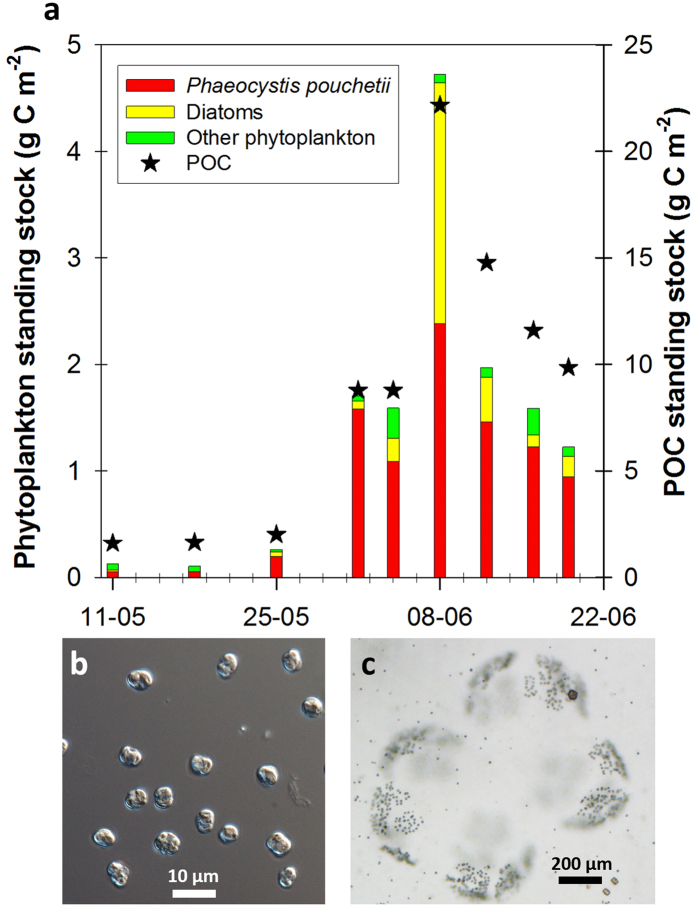
Composition of the under-ice phytoplankton bloom and particulate organic carbon standing stocks. (**a**) Integrated stocks of phytoplankton carbon (coloured bars) with contributions of *Phaeocystis pouchetii*, diatoms and other phytoplankton and particulate organic carbon (black stars) for the upper 50 m surface layer. Micrographs of (**b**), solitary cells (600x magnification) and (**c**), a colony of *P. pouchetii* (100x magnification).

Phytoplankton samples were settled in 50 mL Utermöhl sedimentation chambers (HYDRO-BIOS©, Kiel, Germany) for 48 h. Phytoplankton was identified and enumerated at 100–600× magnification using an inverted Nikon Ti-S light and epifluorescence microscope. The organisms were identified to the lowest taxonomic level possible under inverted light microscopy, ideally to species level, otherwise to genus level or grouped into size-classes. Microscopic counts of the dominant organisms at each depth were always well above the recommended number of 50 per sample. Further, the water column stocks presented in Fig. 2 are integrations of 4 discrete samples from the upper 50 m of the water column, so the total number of specimens counted per predominating species per water column was >100 in most cases, reducing the error to <20%. Randomly chosen individuals of each phytoplankton species/group were measured and the average cell size was used to calculate the biovolume from equivalent geometrical shapes^40^. The biovolume was converted to cellular carbon content using published carbon conversion factors^41^.

Mesozooplankton was sampled with a MultiNet (HYDRO-BIOS©, Kiel, Germany) consisting of five nets with a 0.25 m^2^ opening and 200 μm mesh size at the following depth strata: 0–20, 20–50, 50–200 and 200 m-bottom. Zooplankton were preserved using 4% formaldehyde solution in seawater buffered with hexamethylentetramine and identified to species and stage^42^.

### Irradiance measurements

Solar spectral planar irradiance (E_λ_) was measured simultaneously with two upward-looking Ramses spectral radiometers with cosine collectors (Ramses ACC-VIS, Trios GmbH, Germany). One measured the incident and the second the transmitted irradiance at the bottom of the ice. These measurements were integrated over the wavelength band of photosynthetically active radiation (PAR, 400–700 nm) and then used to estimate the transmittance (fraction of transmitted to incident radiation) of E_PAR_, photosynthetically active radiation (PAR, 400–700 nm) through the ice and snow. The measurements were conducted continuously during floes 3 and 4 at a site representative of the thick snow-covered ice^43^. The same type of sensors were used to determine the transmittance of E_PAR_ for thin ice (<0.25 m) in a refrozen lead. In addition, incident irradiance and irradiance under thick and thin ice was measured with Satlantic HyperOCR hyperspectral radiometers with cosine collectors, at the surface and mounted to a remotely operated vehicle (ROV) respectively. From these measurements, transmittance of E_PAR_ was calculated as with the Ramses data.

### Primary production model

A simple primary production model was applied using photosynthesis versus irradiance data obtained during an Arctic *Phaeocystis*-dominated phytoplankton bloom^15^ combined with measured^16^ and modelled irradiance through thick ice with thick snow cover, thin ice with thin snow cover and open water taking into account the areal fractions of the three different surface types. A detailed description of the primary production model can be found in the Supplementary Information.

### Ice and snow thickness measurements

Total ice and snow thickness was measured with a portable electromagnetic instrument (EM31, Geonics Ltd., Mississauga, Ontario, Canada) mounted on a sledge^44^. In addition, large-scale surveys of total ice and snow thickness were conducted with a helicopter-borne EM instrument (HEM, Ferra Dynamics Inc, Mississauga, Ontario, Canada)^45^. The EM31 and HEM measurements use the same principle. The height above the bottom of the ice is derived from the strength of electromagnetic induction in the conductive sea water under the ice. For the HEM measurements, the height of the instrument above the surface of the ice or snow is determined with a laser altimeter included in the HEM instrument. The EM31 conductivity values were calibrated with drill-hole measurements and post processed to derive total thickness of ice and snow. Snow thickness was measured with a GPS snow probe (Magnaprobe, Snow-Hydro, Fairbanks, AK, USA)^46^. When used together, these two instruments give the spatial distribution of both the total thickness of the ice and snow (from EM31) and the snow depth (from Magnaprobe). For direct comparison of the values, and to subtract’ the snow from the EM31 data, we re-sampled the EM31 data on the Magnaprobe track and applied a Gaussian filter to the EM31 data. The EM31 and Magnaprobe datasets were median-sampled on a 5 m regular grid. Snow depth was subtracted from the EM31 values to derive sea-ice thickness.

## Additional Information

**How to cite this article:** Assmy, P. *et al*. Leads in Arctic pack ice enable early phytoplankton blooms below snow-covered sea ice. *Sci. Rep.* **7**, 40850; doi: 10.1038/srep40850 (2017).

**Publisher's note:** Springer Nature remains neutral with regard to jurisdictional claims in published maps and institutional affiliations.

## Supplementary Material

Supplementary Information

Supplementary Video

## Figures and Tables

**Figure 1:**
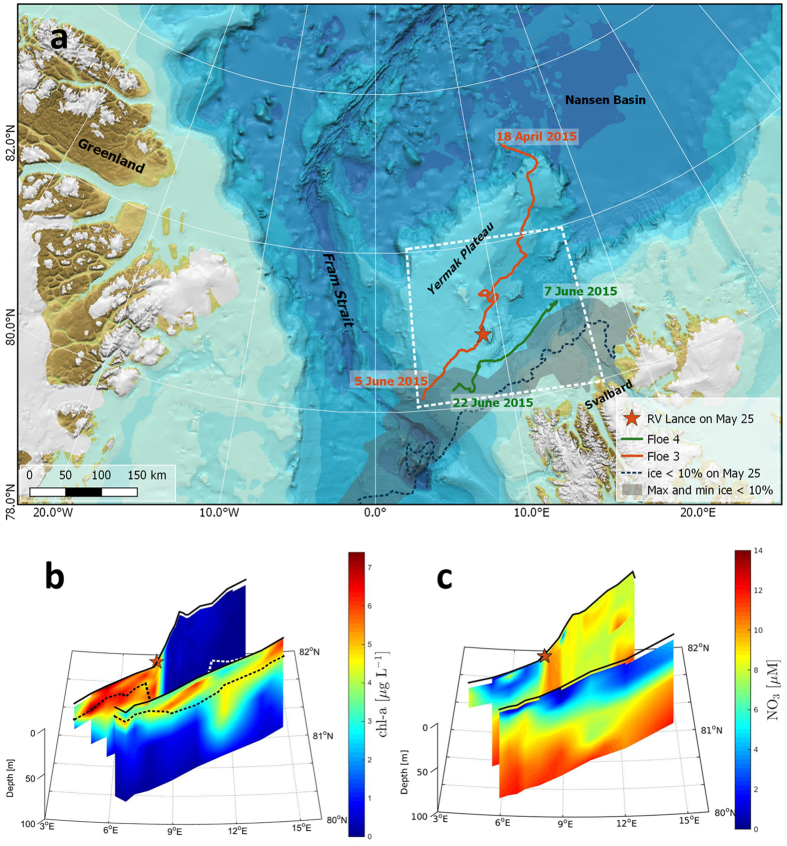
Study location and vertical and spatial extent of the under-ice bloom. (**a**) European Arctic with bathymetry. Orange and green lines are the drift trajectories of floes 3 and 4, respectively, with start and end dates. The location when we first drifted into the under-ice bloom on 25 May is indicated with an orange star. The area demarcating the ice-edge positions between April and June 2015 is shaded in grey. The ice-edge position on 25 May is indicated by the broken blue line and is representative for the bloom period. We define the ice edge as the outer perimeter of a polygon where ice concentration is >10%. The white outline demarcates the area shown in panels b and c. Map created by the Norwegian Polar Institute, Max König with permission from IBCAO^47^. Drift trajectories of floes 3 and 4 showing (**b**) Chlorophyll *a*, and (**c**), nitrate concentrations for the upper 100 m of the water column. The dashed line in (**b**) indicates depth of the pycnocline.

**Figure 3:**
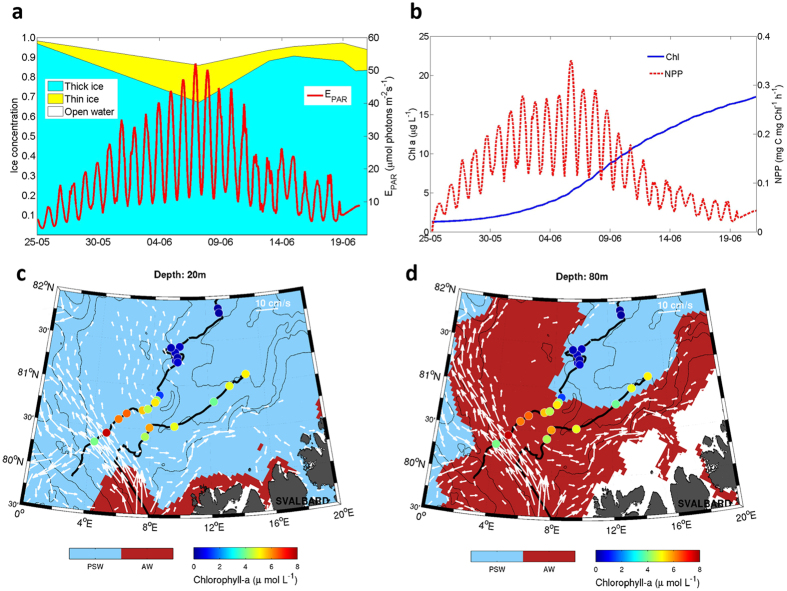
Primary production model and water mass circulation over the Yermak Plateau. (**a**) Open water, thin and thick ice concentration and weight-averaged E_PAR_ right below the sea surface based on the aerial fractions of the three different surface types. The white and coloured areas represent the area fraction of open water and sea ice, respectively, derived from satellite data (Supplementary Fig. S4). E_PAR_ values are modelled from surface E_PAR_ measurements and taking into account the diurnal cycle, different fractions of ice and open water and their respective optical properties. (**b**) Temporal evolution of Chl *a* concentration and net primary production (NPP) during the bloom period predicted by the model. Map of (**c**), surface (20 m) and (**d**), subsurface (80 m) simulated currents from model outputs with currents >2 cm s^−1^. Current velocity is indicated by the size of the vectors (scale on figure). Black lines show drift trajectories. Colour dots show surface Chl *a* concentrations as measured along track indicating the bloom locations. Background colours show surface and subsurface water masses where blue is Polar Surface Water (PSW) and red is Atlantic Water (AW). Areas shallower than 20 m (**c**) and 80 m (**d**) are white. Topography of the Yermak Plateau is shown as thin black lines (500, 1000, 2000 and 3000 m). The maps in (**c**) and (**d**) were generated with the m-map package of Matlab 8.4 (https://www.eoas.ubc.ca/~rich/map.html).

## References

[b1] Arrigo K. R. & van Dijken G. L. Continued increases in Arctic Ocean primary production. Progr. Oceanogr. 136, 60–70 (2015).

[b2] Vancoppenolle M. . Future Arctic Ocean primary productivity from CMIP5 simulations: Uncertain outcome, but consistent mechanisms. Global Biogeochem. Cy. 27, 605–619 (2013).

[b3] Popova E. E. . What controls primary production in the Arctic Ocean? Results from an intercomparison of five general circulation models with biogeochemistry. J. Geophys. Res. 117, C00D12 (2012).

[b4] Michel C., Legendre L., Therriault J. C., Demers S. & Vandevelde T. Springtime coupling between ice algal and phytoplankton assemblages in southeastern Hudson Bay, Canadian Arctic. Polar Biol. 13, 441−449 (1993).

[b5] Strass V. H. & Nöthig E. M. Seasonal shifts in ice edge phytoplankton blooms in the Barents Sea related to the water column stability. Polar Biol. 16, 409−422 (1996).

[b6] Pomeroy L. R. Primary production in the Arctic Ocean estimated from dissolved oxygen. J. Mar. Syst. 10, 1–8 (1997).

[b7] Gosselin M. . New measurements of phytoplankton and ice algal production in the Arctic Ocean. Deep Sea Res. PT II 44, 1623–1644 (1997).

[b8] Fortier M., Fortier L., Michel C. & Legendre L. Climatic and biological forcing of the vertical flux of biogenic particles under seasonal Arctic sea ice. Mar. Ecol. Prog. Ser. 225, 1−16 (2002).

[b9] Arrigo K. R. . Massive phytoplankton blooms under Arctic sea ice. Science 336, 1408–1408 (2012). 22678359 10.1126/science.1215065

[b10] Arrigo K. R. . Phytoplankton blooms beneath the sea ice in the Chukchi Sea. Deep-Sea Res. PT II 105, 1–16 (2014).

[b11] Mundy C. J. . Contribution of under-ice primary production to an ice-edge upwelling phytoplankton bloom in the Canadian Beaufort Sea. Geophys. Res. Lett. 36, L17601 (2009).

[b12] Mundy C. J. . Role of environmental factors on phytoplankton bloom initiation under landfast sea ice in Resolute Passage, Canada. Mar. Ecol. Prog. Ser. 497, 39–49 (2014).

[b13] Granskog M. A. . Arctic research on thin ice: Consequences of Arctic sea ice loss. EOS Trans. AGU 97, 22–26 (2016).

[b14] Willmes S. & Heinemann G. Sea-ice wintertime lead frequencies and regional characteristics in the Arctic, 2003–2015. Remote Sens. 8, doi: 10.3390/rs8010004 (2016).

[b15] Cota G. F., Smith W. O. Jr. & Mitchell B. G. Photosynthesis of *Phaeocystis* in the Greenland Sea. Limnol. Oceanogr. 39, 948–953 (1994).

[b16] Taskjelle T., Granskog M. A., Pavlov A. K., Hudson S. R. & Hamre B. Effects of an Arctic under-ice bloom on solar radiant heating of the water column. J. Geophys. Res. Oceans, doi: 10.1002/2016JC012187 (2016).

[b17] Palmisano A. C. . Photoadaptation in *Phaeocystis pouchetii* advected beneath annual sea ice in McMurdo Sound, Antarctica. J. Plankton Res. 8, 891–906 (1986).

[b18] Sakshaug E. & Skjoldal H. R. Life at the ice edge. Ambio 18, 60–67 (1989).

[b19] Degerlund M. & Eilertsen H. C. Main species characteristics of phytoplankton spring blooms in NE Atlantic and Arctic Waters (68–80 degrees N). Estuaries and Coasts 33, 242–269 (2010).

[b20] Hoppe C. J. M., Holtz L.-M., Trimborn S. & Rost B. Ocean acidification decreases the light-use efficiency in an Antarctic diatom under dynamic but not constant light. New Phytol 207, 159–171 (2015). 25708812 10.1111/nph.13334 PMC4950296

[b21] Rippeth T. P. . Tide-mediated warming of Arctic halocline by Atlantic heat fluxes over rough topography. Nature Geosci. 8, 191–194 (2015).

[b22] Metfies K., von Appen W.-J., Kilias E., Nicolaus A. & Nöthig E.-M. Biogeography and photosynthetic biomass of Arctic marine pico-eukaryotes during summer of the record sea ice minimum 2012. PLoS ONE 11, e0148512, doi: 10.1371/journal.pone.0148512 (2016). 26895333 PMC4760976

[b23] Ray J. L. . Molecular gut content analysis demonstrates that *Calanus* grazing on *Phaeocystis pouchetii* and *Skeletonema marinoi* is sensitive to bloom phase but not prey density. Mar. Ecol. Prog. Ser. 542, 63−77 (2016).

[b24] Reigstad M., Wexels Riser C., Wassmann P. & Ratkova T. Vertical export of particulate organic carbon: Attenuation, composition and loss rates in the northern Barents Sea. Deep Sea Res. PT II 55, 2308–2319 (2008).

[b25] Wassmann P., Vernet M., Mitchell B. G. & Rey F. Mass sedimentation of *Phaeocystis pouchetii* in the Barents Sea. Mar. Ecol. Prog. Ser. 66, 183–195 (1990).

[b26] Reigstad M. & Wassmann P. Does *Phaeocystis* spp. contribute significantly to vertical export of organic carbon? Biogeochem. 83, 217–234 (2007).

[b27] Søreide J. E. . Sympagic-pelagic-benthic coupling in Arctic and Atlantic waters around Svalbard revealed by stable isotopic and fatty acid tracers. Mar. Biol. Res. 9, 831–850 (2013).

[b28] Le Moigne F. A. C. . Carbon export efficiency and phytoplankton community composition in the Atlantic sector of the Arctic Ocean. J. Geophys. Res. 120, 3896–3912 (2015).

[b29] Lalande C., Bauerfeind E. & Nothig E.-M. Downward particulate organic carbon export at high temporal resolution in the eastern Fram Strait: influence of Atlantic Water on flux composition. Mar. Ecol. Prog. Ser. 440, 127–136 (2011).

[b30] Kwok R., Spreen G. & Pang S. Arctic sea ice circulation and drift speed: Decadal trends and ocean currents. J. Geophys. Res. 118, 2408–2425 (2013).

[b31] Bintanja R. & Selten F. M. Future increases in Arctic precipitation linked to local evaporation and sea-ice retreat. Nature 509, 479–484 (2014). 24805239 10.1038/nature13259

[b32] Carmack E. . Towards quantifying the increasing role of oceanic heat in sea ice loss in the new Arctic. B. Am. Meteorol. Soc. 97, 2079–2105 (2015).

[b33] Lasternas S. & Agusti S. Phytoplankton community structure during the record Arctic ice-melting of summer 2007. Polar Biol. 33, 1709–1717 (2010).

[b34] Lalande C., Bauerfeind E., Nöthig E.-M. & Beszczynska-Moller A. Impact of a warm anomaly on export fluxes of biogenic matter in the eastern Fram Strait. Progr. Oceanogr. 109, 70–7 (2013).

[b35] Nöthig E.-M. . Summertime plankton in Fram Strait – a compilation of long- and short-term observations. Polar Res. 34, 23349 (2015).

[b36] Knap A., Michaels A., Close A., Ducklow H. & Dickson A. Measurement of Chlorophyll a and Phaeopigments by fluorometric analysis. JGOFS Rep. 19, 118–122 (1996).

[b37] Dickson A. G., Sabine C. L. & Christian J. R. Guide to best practices for ocean CO_2_ measurements. PICES Spec. Publ. 3, 191 pp (2007).

[b38] Assmy P. . N-ICE2015 water column biogeochemistry (v1.0) [Data set]. Norwegian Polar Institute. doi: 10.21334/npolar.2016.3ebb7f64 (2009).

[b39] Nymark M. . An integrated analysis of molecular acclimation to high light in the marine diatom *Phaeodactylum tricornutum*. PloS ONE 11, e7743 (2009). 10.1371/journal.pone.0007743 PMC2766053 19888450

[b40] Hillebrand H., Duerselen C. D., Kirschtel D., Pollingher U. & Zohary T. Biovolume calculation for pelagic and benthic microalgae. J Phycol 35, 403–424 (1999).

[b41] Menden-Deuer S. & Lessard E. J. Carbon to volume relationships for dinoflagellates, diatoms and other protist plankton. Limnol. Oceanogr. 45, 569–579 (2000).

[b42] Kwasniewski S., Hop H., Falk-Petersen S. & Pedersen G. Distribution of *Calanus* species in Kongsfjorden, a glacial fjord in Svalbard. J. Plankton. Res. 25, 1–20 (2003).

[b43] Taskjelle T., Hudson S. R., Pavlov A. & Granskog M. A. N-ICE2015 surface and under-ice spectral shortwave radiation data (v1.4) [Data set]. Norwegian Polar Institute, doi: 10.21334/npolar.2016.9089792e (2016).

[b44] Rösel A. . N-ICE2015 total (snow and ice) thickness data from EM31 (v1.0) [Data set]. Norwegian Polar Institute. doi: 10.21334/npolar.2016.70352512 (2016).

[b45] King J., Gerland S., Spreen G. & Bratrein M. N-ICE2015 sea-ice thickness measurements from helicopter-borne electromagnetic induction sounding [Data set]. Norwegian Polar Institute. doi: 10.21334/npolar.2016.aa3a5232 (2016).

[b46] Rösel A. . N-ICE2015 snow depth data with Magna Probe [Data set]. Norwegian Polar Institute. doi: 10.21334/npolar.2016.3d72756d (2016).

[b47] Jakobsson M. . The International Bathymetric Chart of the Arctic Ocean (IBCAO) Version 3.0. Geophys. Res. Lett. 39, L12609 (2012).

