# Supplementary material for: Leads in Arctic pack ice enable early phytoplankton blooms below snow-covered sea ice
Source: Sci Rep. 2017 Jan 19;7:40850. doi: 10.1038/srep40850 (PMC5244362; doi:10.1038/srep40850)
Supplement: Supplementary Information [file srep40850-s1.doc]

**Supplementary Information**

**Leads in Arctic pack ice enable early phytoplankton blooms below snow-covered sea ice**

Philipp Assmy^1^, Mar Fernández-Méndez^1^, Pedro Duarte^1^, Amelie Meyer^1^, Achim Randelhoff^1,2^, Christopher J. Mundy^3^, Lasse M. Olsen^1^, Hanna M. Kauko^1^, Allison Bailey^1^, Melissa Chierici^4^, Lana Cohen^1^, Anthony P. Doulgeris^5^, Jens K. Ehn^3^, Agneta Fransson^1^, Sebastian Gerland^1^, Haakon Hop^1,2^, Stephen R. Hudson^1^, Nick Hughes^6^, Polona Itkin^1^, Geir Johnsen^7,8^, Jennifer A. King^1^, Boris P. Koch^9^, Zoe Koenig^10^, Slawomir Kwasniewski^11^, Samuel R. Laney^12^, Marcel Nicolaus^9^, Alexey K. Pavlov^1^, Christopher M. Polashenski^13^, Christine Provost^10^, Anja Rösel^1^, Marthe Sandbu^7^, Gunnar Spreen^1,14^, Lars H. Smedsrud^15,16^, Arild Sundfjord^1^, Torbjørn Taskjelle^17^, Agnieszka Tatarek^11^, Jozef Wiktor^11^, Penelope M. Wagner^6^, Anette Wold^1^, Harald Steen^1^ and Mats A. Granskog^1^

^1^Norwegian Polar Institute, Fram Centre, 9296 Tromsø, Norway

^2^Department of Arctic and Marine Biology, Faculty of Biosciences, Fisheries and Economics, UiT The Arctic University of Norway, 9037 Tromsø, Norway

^3^Centre for Earth Observation Science, University of Manitoba, Winnipeg, MB R3T 2N2, Canada

^4^Institute of Marine Research, 9019 Tromsø, Norway

^5^Department of Physics and Technology, Faculty of Science and Technology, UiT The Arctic University of Norway, 9037 Tromsø, Norway

^6^Norwegian Meteorological Institute, 9239 Tromsø, Norway

^7^Centre for Autonomous Marine Operations and Systems, Department of Biology, Norwegian University of Science and Technology, 7491 Trondheim, Norway

^8^ University Centre in Svalbard, Post box 156, 9171 Longyearbyen, Norway

^9^Alfred Wegener Institute, Helmholtz Center for Polar and Marine Research, 27570 Bremerhaven, Germany

^10^LOCEAN, UMR 7159, CNRS/UPMC/MNHN/IRD, Pierre and Marie Curie University, Paris cedex, France

^11^Institute of Oceanology, Polish Academy of Sciences, 81-712 Sopot, Poland

^12^Biology Department, Woods Hole Oceanographic Institution, Woods Hole, MA 02543, USA

^13^U.S. Army, Cold Regions Research and Engineering Laboratory, Hanover, NH 03755, USA

^14^Institute of Environmental Physics, University of Bremen, 28334 Bremen, Germany

^15^Bjerknes Centre for Climate Research, 5007 Bergen, Norway

^16^Geophysical Institute, University of Bergen, 5007 Bergen, Norway

^17^Department of Physics and Technology, University of Bergen, 5007 Bergen, Norway

**Author Information. Correspondence and requests for materials should be addressed to P.A. (Philipp.Assmy@npolar.no).**

1. **Upper ocean stratification and turbulent mixing**

During the N-ICE2015 expedition, upper-ocean microstructure profiler data were collected several times per day. Based on high resolution shear measurements, temperature and salinity data, the eddy diffusivity can be derived by assuming a set dissipation ratio coefficient and a balance between the production of turbulent kinetic energy, the dissipation, and the buoyancy flux^1,2^. Prior to the bloom period, the pycnocline was deep and the stratification, which is diagnosed with the buoyancy frequency, was weak (Table S1). As the bloom developed, the upper part of the water column became fresher due to addition of sea ice melt water; stratification increased and the pycnocline became shallower (Table S1). Mixing, which is quantified as eddy diffusivity, was lower during the initial phase of the bloom (Table S1). A storm during floe 4 led to increased mixing but constant ice melt kept the upper water column well stratified.

**Table S1 |** Mean oceanographic properties from microstructure profiler data prior to the bloom (11–24 May) and during the bloom on floe 3 (26 May–3 June) and floe 4 (7–18 June). Accuracy for the conductivity estimate is ± 0.0003 Sm^-1^, ±0.001^o^C for temperature, and ±1m for pycnocline depth. Noise level for buoyancy frequency is 3 x 10^-6^ s^-2^ and 1 x 10^-5^ m^2^s^-1^ for eddy diffusivity.

| Bloom stage | Conservative temperature upper 10 m | Absolute Salinity  upper 10 m | Buoyancy frequency  upper 50 m | Eddy diffusivity upper 50 m | Pycnocline depth (m) |
| --- | --- | --- | --- | --- | --- |
| Prior to bloom | -1.84 ^o^C | 34.45 gkg^-1^ | 0.02x10^-4^ (rads^-1^)^2^ | 3x10^-3^ m^2^s^-1^ | 98 |
| Bloom floe 3 | -1.77 ^o^C | 34.03 gkg^-1^ | 0.6x10^-4^ (rads^-1^)^2^ | 1x10^-3^ m^2^s^-1^ | 16 |
| Bloom floe 4 | -1.23 ^o^C | 33.52 gkg^-1^ | 1.7x10^-4^ (rads^-1^)^2^ | 5x10^-3^ m^2^s^-1^ | 21 |

**References**

1. Osborn, T.R. Estimates of the local rate of vertical diffusion from dissipation measurements. *J. Phys. Oceanogr.* **10,** 83-89 (1980).
2. Fer, I. Scaling turbulent dissipation in an Arctic fjord. *Deep-Sea Res. PT II* **53,** 77–95 (2006).
3. **Protist plankton composition**

**Table S2 |** List of protist plankton taxa identified with inverted light microscopy in samples from 5 m depth during the bloom period (25 May – 22 June 2015). Taxonomic ranks according to World Register of Marine Species (WoRMS).

**Diatoms**

*Attheya septentrionalis*

Centric diatoms <30um

*Ceratoneis closterium*

*Chaetoceros atlanticus*

*Chaetoceros borealis*

*Chaetoceros decipiens*

*Chaetoceros furcellatus*

*Chaetoceros simplex*

*Chaetoceros socialis*

*Chaetoceros* sp.

*Chaetoceros tenuissimus*

*Conticribra weissflogii*

*Entomoneis kjellmanii* var. *kariana*

*Eucampia groenlandica*

*Fragilariopsis cylindrus*

*Fragilariopsis oceanica*

*Hantzschia weyprechtii*

*Navicula* cf. *gelida*

*Navicula distans*

*Navicula* sp. >30um

*Navicula vanhoeffenii*

*Nitzschia frigida*/*neo-frigida*

*Nitzschia* sp.

Pennate diatoms <30um

*Pseudo-nitzschia delicatissima*/*pseudodelicatissima*

*Pseudo-nitzschia granii*

*Pseudo-nitzschia seriata*

*Pseudo-nitzschia* sp.

*Pseudo-nitzschia* cf. *granii*

*Skeletonema costatum*

*Thalassiosira bioculata*

*Thalassiosira gravida/antarctica*

*Thalassiosira nordenskioeldii*

*Thalassiosira* sp.

**Dinoflagellates**

*Alexandrium* sp.

*Amphidinium crassum*

*Azadinium spinosum*

*Cochlodinium pulchellum*

Dinoflagellates indet. cyst

*Gonyaulax* sp.

*Gymnodinium arcticum*

*Gymnodinium galeatum*

*Gymnodinium gracilentum*

*Gymnodinium simplex*

*Gymnodinium* sp. 10-20um

*Gymnodinium* sp. 7-10um

*Gymnodinium* cf. *wulffii*

*Gyrodinium* cf. *spirale*

*Gyrodinium* *flagellare*

*Gyrodinium fusiforme*

*Gyrodinium* sp. 20-30um

*Heterocapsa rotundata*

*Heterocapsa* sp.

*Heterocapsa* cf. *niei*

*Katodinium glaucum*

*Lessardia elongata*

*Oxyrrhis* sp.

*Polarella glacialis*

*Pronoctiluca pelagica*

*Prorocentrum minimum*

*Protoperidinium bipes*

*Protoperidinium pellucidum*

*Protoperidinium* sp.

**Prymnesiophytes**

*Algirosphaera robusta*

Coccolithophores indet. 6-10um

*Emiliania huxleyi*

*Phaeocystis pouchetii*

Prymnesiophytes indet. 7-10um

**Ciliates**

Ciliates indet.

Ciliates indet. 10-20 µm

*Laboea strobila*

*Leegaardiella sol*

*Lohmanniella oviformis*

*Mesodinium rubrum*

Oligotrich ciliates indet.

*Strombidium* sp.

*Uronema marinum*

**Prasinophytes**

Prasinophytes indet. 6-10um

*Pyramimonas* sp.

*Pyramimonas* cf. *nansenii*

*Pyramimonas* cf. *virginica*

**Cryptophytes**

Cryptophytes indet.

*Plagioselmis* sp.

*Teleaulax acuta*

*Teleaulax amphioxeia*

*Teleaulax* sp.

*Telonema subtile*

Cryptophytes incertae sedis

*Leucocryptos marina*

**Choanoflagellates**

*Bicosta spinifera*

Choanoflagellates indet.

*Monosiga marina*

**Chrysophytes**

*Dinobryon faculiferum*

*Dinobryon* sp.

**Flagellates indetermined**

Bifagellates indet. 7-10um

Biflagellates indet. 3-7um

Biflagellates indet. ~3um

Flagellates indet. ~3um

Flagellates indet. 3-7um

Flagellates indet. 7-10um

Flagellates indet. 11-15um

**Chlorophytes**

Chlorophytes indet.

**Euglenozoa**

Euglenoidea indet.

**Incertae sedis**

*Commation cryoporinum*

**Uncertain taxa**

Coccoid cells

1. **Dissolved inorganic carbon**


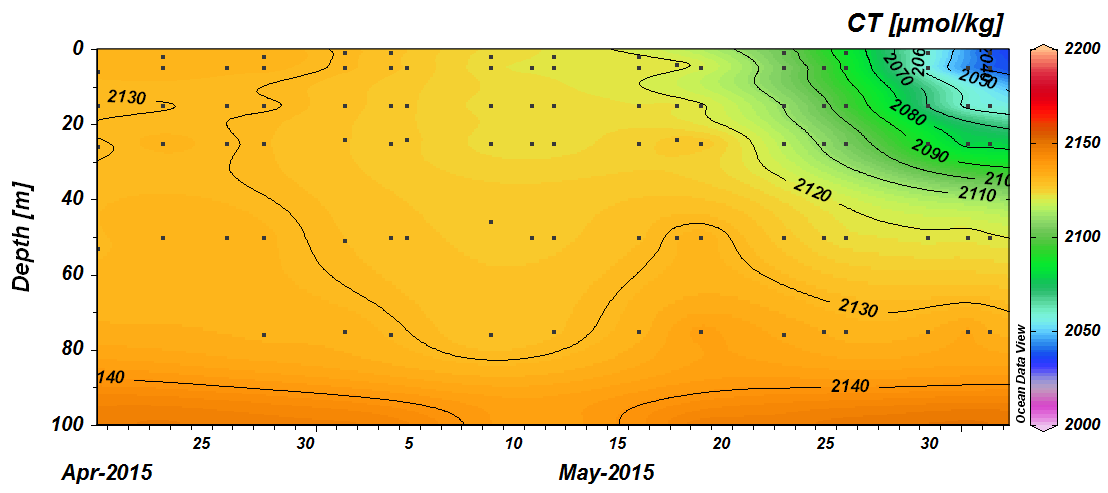


**
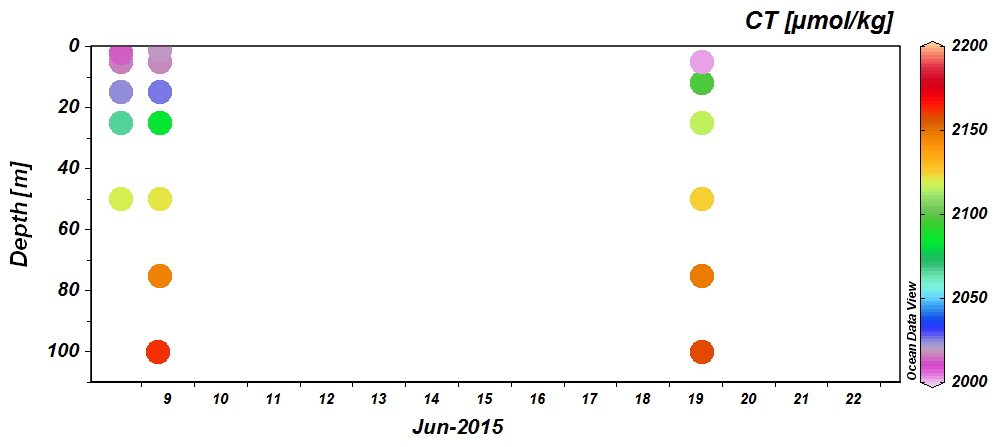
**

**Figure S1 |** Dissolved inorganic carbon (DIC) concentrations (in µmol kg^-1^) for the upper 100m for floes 3 (upper panel) and 4 (lower panel). Note that DIC was only sampled at three stations during drift of floe 4 and that the scales of the date axes are different in the two panels. All DIC data were salinity-normalized and, thus, the decline in DIC is due to biological uptake and not freshening. Figure S1 was generated with Ocean Data View version 4.7.8 ([odv.awi.de](http://odv.awi.de/))^1^.

**References**

1. Schlitzer, R., Ocean Data View. [odv.awi.de](http://odv.awi.de/) (2015).
2. **Sea ice and snow thickness distribution**

**
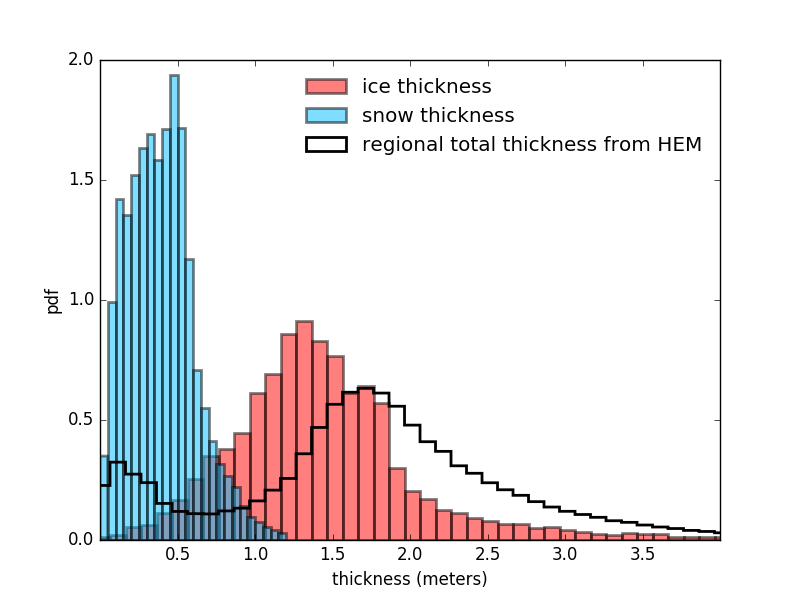
**

**Figure S2 |** Total (ice and snow) thickness distribution representative of the regional scale (tens of km’s) measured with a helicopter-borne electromagnetic instrument (HEM, black line), illustrating the thin and thick ice mode and probability density function (PDF). Additionally, the PDF of snow (blue histogram) and ice (red histogram) thickness from floes 3 and 4 are shown. Snow and ice thickness are derived from a portable electromagnetic instrument (EM31) and a GPS snow probe.

1. **Sea Ice conditions**

The sea ice conditions in the larger vicinity (~100 km) of the ship were observed by Synthetic Aperture Radar (SAR) satellite data (sections 5.1 and 5.2) and by a buoy array measuring sea ice drift and deformation (section 5.3). These observations allow quantification of lead fraction during the period when the phytoplankton bloom was observed in the region.

**5.1 Leads**

SAR satellite images from RADARSAT-2 in ScanSAR Wide mode with a resolution of about 100 m were available on a near daily basis throughout the expedition (not shown). Figure S3 shows an example for 27 May 2015. The two polarization channels HH and HV were analysed to create an RGB-composite, where blue areas represent open water and leads with thin ice. At the time of the image acquisition, a system of large leads (several hundred meters wide and tens of kilometres long) had opened up around the ship and towards the ice margin (green arrows in Fig. S3). These leads allowed light to penetrate directly into the ocean.

To estimate the amount of open water and thin ice, higher resolution quad-pole images from RADARSAT-2 and ALOS-2 PALSAR 2 were acquired from 24 May–21 June to provide a more detailed view of sea ice conditions in the vicinity of RV Lance (section 5.2). Two of these scenes are marked in Supplementary Fig. S3 with red boxes.

Wide-swath SAR images were not used in this case, because of unresolved incidence angle and noise-floor issues that have not yet been accounted for in the automatic analysis. Furthermore, the extra information in quad-pol images makes the classification decision more confident, and the higher spatial resolution (~5x10 m per pixel) enabled resolving finer scale leads and presumably more accurate lead fractions


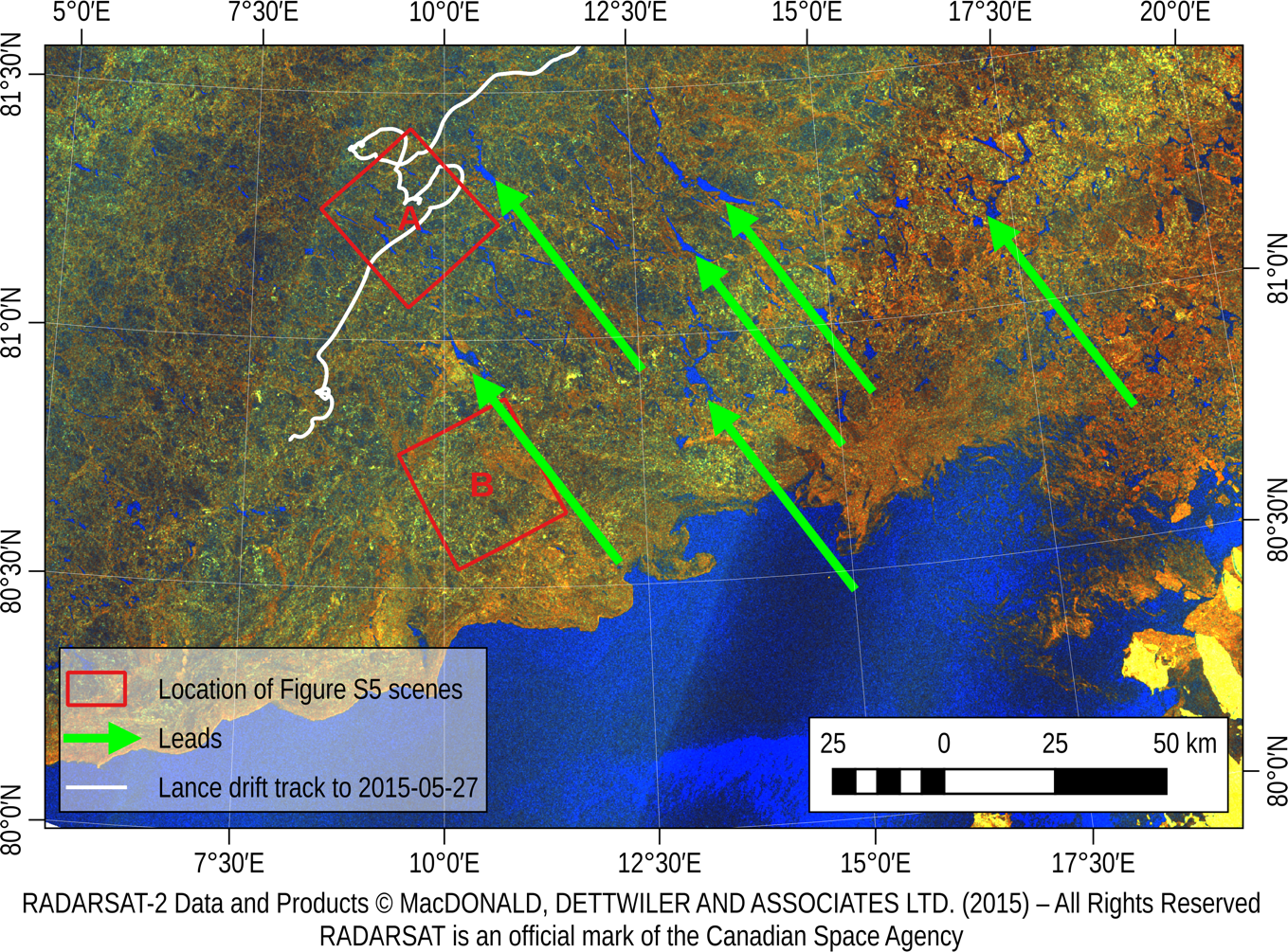
**Figure S3 |** RADARSAT-2 image in ScanSAR Wide mode from 27 May 2015 06:30 UTC rendered as an RGB-composite (R=HH, G=HV and B=HV/HH) showing leads (green arrows) in the vicinity of our study area and the locations of the 2 satellite images in Supplementary Figure S3 (A = 24 May 2015, and B = 15 June 2015).

**5.2 Ice type classification**

High resolution RADARSAT-2 (Fine Quad-Pol mode, hereafter RS-2) and ALOS-2 PALSAR-2 (Stripmap Full Quad-Pol, hereafter ALOS-2) scenes were analysed using an automatic segmentation algorithm developed at UiT The Arctic University of Norway that separated areas with similar statistical properties into distinct segments^1,2^. These segments were subsequently labelled by ice analysts to obtain classified images, and the percentage fraction of each segment was then determined. The areal fractions corresponding to open water, thin ice and thick ice were then used in the modelling of transmitted light (see section 6 below).


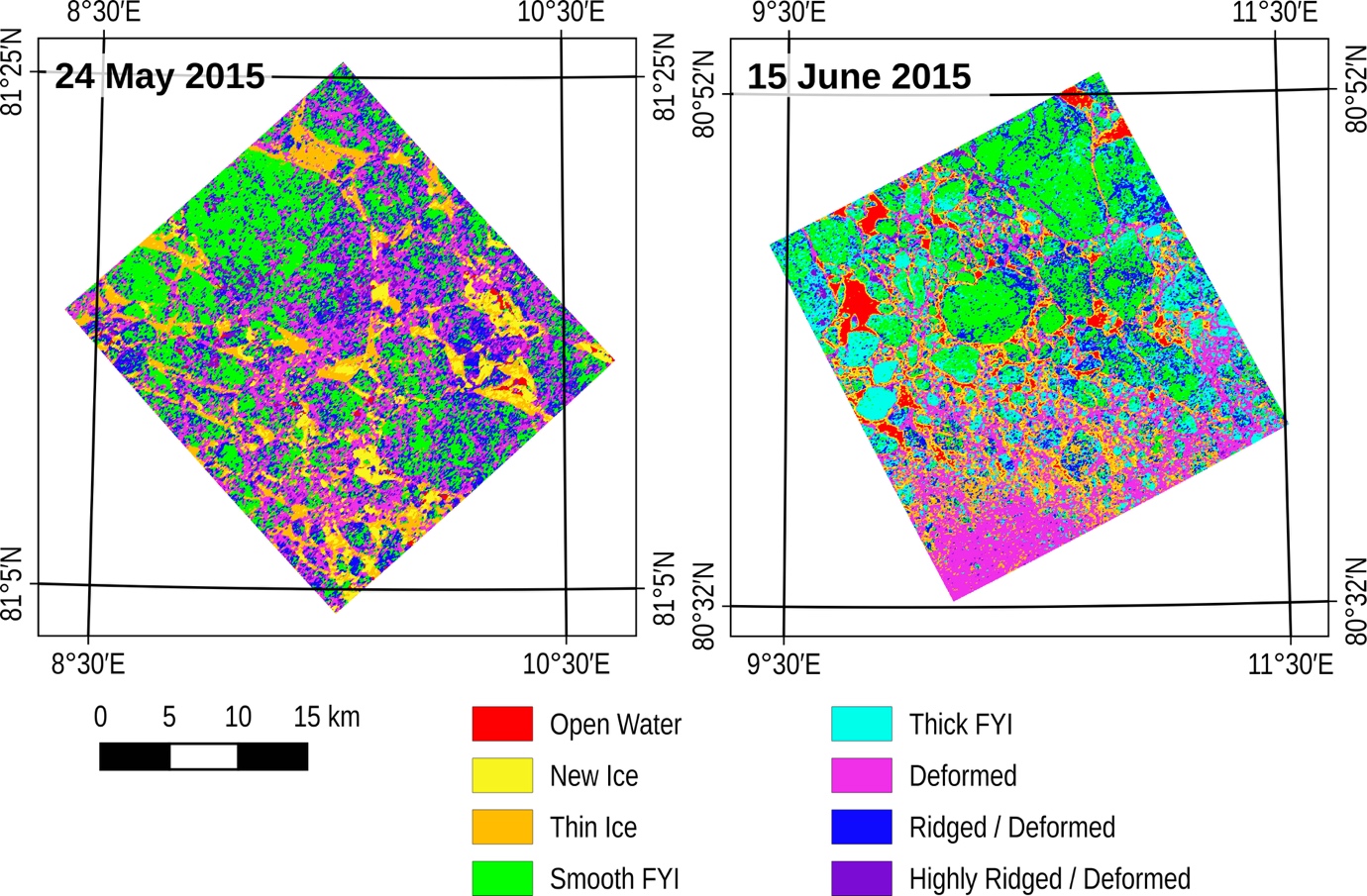


**Figure S4 |** Ice type classification based on RS-2 images for 24 May (left panel) and 15 June (right panel) 2015. The segments were automatically calculated, and then manually identified by ice analysts and the thin ice classes (open water, new ice, and thin ice) used to derive the lead fraction. The software used for Figure S4 was QGIS (<http://www.qgis.org/>), version 2.8 "Wien".

The segment decision is based on both polarimetric and textural information, and all pixels with similar statistical properties are grouped in the same cluster^1,2^. The analysis was performed on seven quad-pole scenes that were reasonably closely located to the ship at time of acquisition (Table S3) with a large window size of 18x18 pixels for RS-2 and 27x27 pixels for ALOS-2 and a moderate sensitivity to obtain few and reasonably smooth generic segments that can then be identified by ice analysts.

The analysis on these segments was conducted by researchers at the Norwegian Ice Service (NIS) following procedures used by all ice analysts at operational services documented by the Canadian Ice Service in MANICE^3^. The intrinsic knowledge of the area in which the ice service specializes in is also used. Based on the specific segmentation parameters for individual SAR images in May-June 2015, there was an overall good contrast with areas of open water and lead features (with either open water or thin ice formations) and some areas where thicker ice floes were well defined. The aerial fractions are shown in Fig 3a and for simplicity values were linearly interpolated between subsequent scenes.

Smooth ice floes were identified by distinct polygonal shapes with sharp boundaries approximately >150 m and relatively low backscatter compared to surrounding linear features^4^. Lead and ridging features have distinct linear patterns but normally differ in brightness, width and contours that are characteristic to both structures. Leads are shown to have wide areas of low backscatter between two straight sharp boundaries. Low backscatter areas are either open water or new ice formations but can clearly be seen developing in several stages in these large areas between leads. Raised sea ice edges (tens of cm) and ridges can either follow a straight trajectory or a slightly winding pattern due to being formed in areas of sea ice floe convergence^5,6^. New ice forming at lead edges was very well defined because it followed the lead contours. Additionally, the development of nilas could be seen clearly, particularly within lead areas.

**Table S3 |** Quad-pol SAR satellite images over a month from May 24- June 21 2015 were used to provide supplementary information on sea ice conditions for the area.

| Name | Satellite | Date | Time | Mode |
| --- | --- | --- | --- | --- |
| RS2_20150524 | RADARSAT-2 | 2015-05-24 | 14:37:57.821533Z | FQ4 |
| AP2_20150607a | ALOS-2 PALSAR-2 | 2015-06-07 | 21:19:46.541000Z | HBQR1.1 |
| RS2_20150613 | RADARSAT-2 | 2015-06-13 | 14:54:27.741577Z | FQ8 |
| RS2_20150615 | RADARSAT-2 | 2015-06-15 | 15:36:12.906189Z | FQ15 |
| RS2_20150619 | RADARSAT-2 | 2015-06-19 | 15:19:31.401611Z | FQ6 |
| RS2_20150620 | RADARSAT-2 | 2015-06-20 | 16:30:30.420891Z | FQ24 |
| RS2_20150621 | RADARSAT-2 | 2015-06-21 | 16:01:14.290222Z | FQ16 |

**5.3 Sea ice deformation**

The amount and distribution of leads (open water and thin ice, i.e, high light transmission), adjacent young ridges (thick deformed ice close to leads, i.e., medium light transmission) and older level or ridged sea ice (ice covered by thick snow, i.e. low light transmission) in a certain area will depend on the sea ice drift and especially its deformation. In a divergent ice drift regime leads will open up while for ice convergence the ice cover will close and the ice will raft or get ridged.

Sea ice divergence and drift until 5 June were measured by an array of GPS sea ice drifters that were deployed on floe 3 in a circular array up to 40 km away from the ship^7^. After relocation of the ship to floe 4 and until the end of the time series, sea ice deformation data are no longer available and the sea ice drift vectors are taken from the passive drift of the ship. Sea ice divergence was calculated by the Green method^8^ from line integrals along triangles formed between positions of 14 buoys in the array. Divergence values represent the mean of all triangles of the buoy array. Sea ice concentration values in the time series are for a 43.75 km^2^ grid box centred around the ship and are based on AMSR2 ASI sea ice concentrations^9,10^.

The dominant ice drift is directed southward with highest drift speeds during the storm events^11^ (shaded areas in Fig. S5) which also caused the peak values in divergence. Positive divergence values are associated with the opening of the ice pack and lead development. This results in a reduction of sea ice concentrations that slightly lag the peaks in divergence (see for example 17 May). Negative divergence (convergence) events close the ice pack and cause subsequent rafting and ridging processes. The RADARSAT-2 shown in Fig. S3 (timing indicated by grey vertical bar in Fig. S5) was taken during a period of slower sea ice drift in-between two storms. The ice was oscillating between divergent and convergent regimes on sub-daily time scales during that period. Figure S3 shows a situation of a particularly well developed lead system compared to the surrounding days, not untypical for the overall conditions during the N-ICE2015 expedition.

During and after the storm starting on 1 June, atmospheric temperatures rose above the freezing point of sea water^11^. This is reflected in an increase of the sea ice drift speed and decline in the sea ice concentration as a result of sea ice melt and more open water between the individual sea ice floes and wind-driven ice export out of the area. The correspondence of the open water fraction estimated from SAR data (stars in Fig. S5; open water fraction is the remainder after subtracting thick and thin ice from 100% as in Fig 3a) and sea ice concentration is good, given the fact that the former was obtained by classifying SAR images that did not necessarily fall within the grid box for which sea ice concentration was averaged. For the period from 4 May–22 June, an interrupted but regular presence of open water and thin ice with little snow cover that allowed, in contrast to thick snow-covered ice, relatively high light transmission into the underlying water column and facilitated the *in situ* development of the under-ice phytoplankton bloom.

**
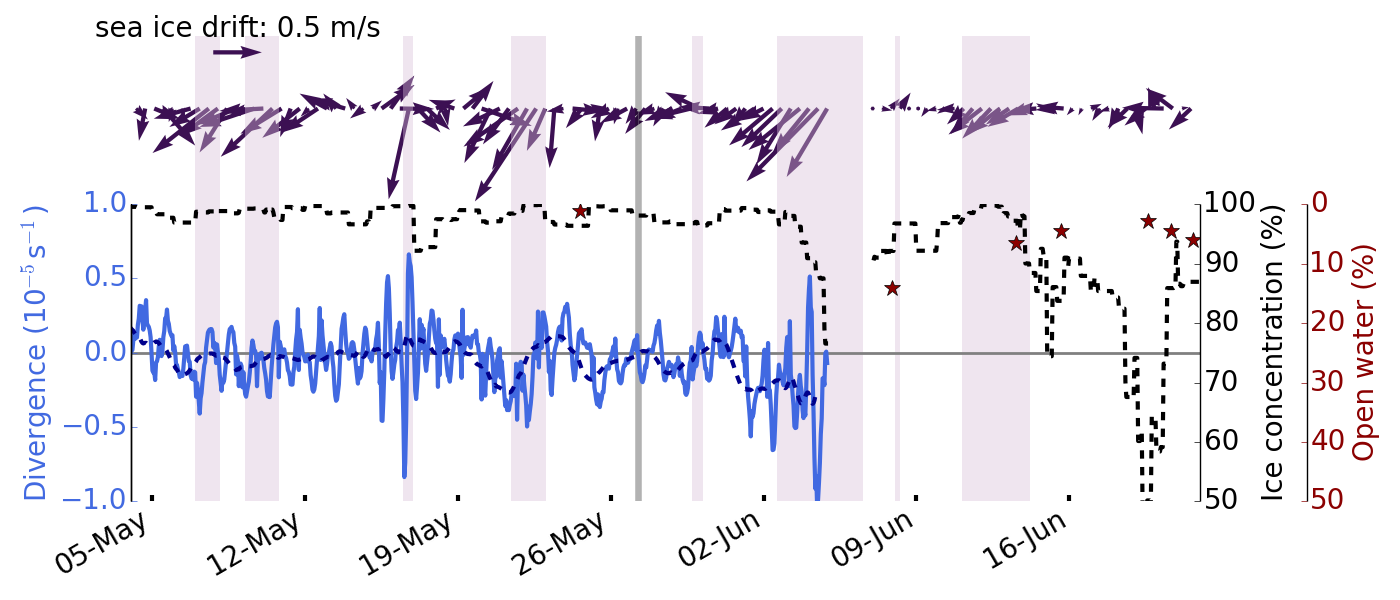
**

**Figure S5 |** Sea ice divergence (hourly resolved data in solid blue line, 24 h running mean in dashed dark blue), sea ice concentration (dashed black line), and sea ice drift vectors for the period 4 May–22 June 2015. The duration of storm events (winds stronger than 8 m s^-1^ lasting more than 3 h with no breaks more than 1 h) is marked by shading. Open water fraction measured by the classified SAR images are marked by red stars. The grey vertical line marks the date of the SAR acquisition shown in Fig. S3.

**References**

1. Doulgeris, A.P. & Eltoft, T. Scale mixture of Gaussian modelling of polarimetric SAR data. *EURASIP J. Appl. Signal Proc*. **2010**, ID 874592 (2010).
2. Doulgeris, A.P. A Simple and Extendable Segmentation Method for Multi-Polarisation SAR Scenes. *Proc. POLinSAR 2013*, Frascati, Italy, 8 pp., 28 January - 1 February, 2013 (2013).
3. Environment Canada. Manual of Standard Procedures for Observing and Reporting Ice Conditions (MANICE). Issuing authority: Assistant Deputy Minister, Meteorological Service of Canada: <http://www.ec.gc.ca/glaces-ice/default.asp?lang=En&n=08D7D137-1> (2005).
4. Dierking, W. Sea ice classification on different spatial scales for operational and scientific use / L. Ouwehand (editor), In: Proceedings of ESA Living Planet Symposium, 9-13 September 2013, Edinburgh, UK, (ESA SP; 722), Noordwijk, The Netherlands, European Space Agency (ESA), ISBN: 978-92-9221-286-5 (2013).
5. Shokr, M. & Sinha, N. Retrieval of Sea Ice Surface Features, in Sea Ice: Physics and Remote Sensing, John Wiley & Sons, Inc, Hoboken, NJ. doi:10.1002/9781119028000.ch9 (2015).
6. Dierking, W. & Dall, J. Sea-ice deformation state from synthetic aperture radar imagery. Part II: effects of spatial resolution and noise level. *IEEE Trans. Geosci. Remote Sens.* **45**, 2197–2207 (2008).
7. Itkin, P., *et al.* N-ICE2015 buoy data [Data set]. Norwegian Polar Institute. <https://doi.org/10.21334/npolar.2015.6ed9a8ca> (2015).
8. Hutchings, J.K., Roberts, A., Geiger, C.A., Richter-Menge, J. Spatial and temporal characterization of sea-ice deformation, *Ann. Glaciol.* **52,** 360-368 (2011).
9. AMSR2 sea ice concentration on a 6.25 km grid, ASI algorithm, ver. 5.2, University of Bremen, <https://seaice.uni-bremen.de/amsr2/>.
10. Spreen, G., Kaleschke, L. & Heygster, G. Sea ice remote sensing using AMSR-E 89 GHz channels, J. Geophys. Res. 113, C02S03 (2008).
11. Hudson, S. R., Cohen, L., & Walden, V. N-ICE2015 surface meteorology v2 [Data set]. Norwegian Polar Institute. doi:10.21334/npolar.2015.056a61d1 (2015).

**6. Aggregate light field and primary production estimate**

**6.1 Light parameterization**

Radiative transfer modeling, using AccuRT^1^, a plane-parallel coupled atmosphere-ocean radiative transfer model based on DISORT^2^, was done for each of the three major surface types^3^ (thick ice with snow, thin ice -representing refrozen leads- and open water). Vertical profiles of planar and scalar irradiance were calculated. Inherent optical properties (IOP) in the upper 20 m of the water column, measured with a WetLabs ac-9^4^, were used as input, and the Henyey-Greenstein scattering phase function with asymmetry factor 0.92 applied.

A simulation was performed for each of the 34 profiles collected with the ac-9 during the N-ICE2015 expedition, using vertically averaged values of the IOPs from the upper 20m.

Measured spectral irradiance incident on, and transmitted through, thick ice^4^ with snow and thin ice was used to adjust the properties of the ice and snow in the model. The model results were used to obtain a time series (Fig. S6) of the ratio of scalar to downwelling planar irradiance (Eo:Ed ) right underneath the ice or sea surface (in case of open water) for photosynthetically active radiation (EPAR; 400-700 nm). These ratios are used to estimate Eo in the water column, based on available Ed data for the primary production model (see section 6.2 below). The mean Eo:Ed ratios were 1.282 ±0.031 for open water, 1.835 ±0.049 for thin ice and 1.858 ±0.050 for thick ice.

**References**

1. Hamre, B., Stamnes, S., Stamnes, J.J. & Stamnes, K. AccuRT: A Versatile Tool for Radiative Transfer in Coupled Media like Atmosphere-Ocean Systems. In /Ocean Optics XXII/. Portland, ME, USA (2014). <http://www.geminor.com/media/Hamre_OO2014_A0_portrait_v3.pdf>.
2. Thomas, G.E. & Stamnes, K. Radiative Transfer in the Atmosphere and Ocean. Cambridge: Cambridge University Press (1999).
3. Taskjelle, T., Granskog, M.A., Pavlov, A.K., Hudson, S.R. & Hamre, B. Effects of an Arctic under-ice bloom on solar radiant heating of the water column, *J. Geophys. Res. Oceans*, doi:10.1002/2016JC012187 (in press).
4. Taskjelle, T., Granskog, M. A., Pavlov, A., Hudson, S. R., & Hamre, B. N-ICE2015 total attenuation and absorption profiles from water column with AC-9 [Data set]. Norwegian Polar Institute. doi:10.21334/npolar.2016.114bfaaa (2016).


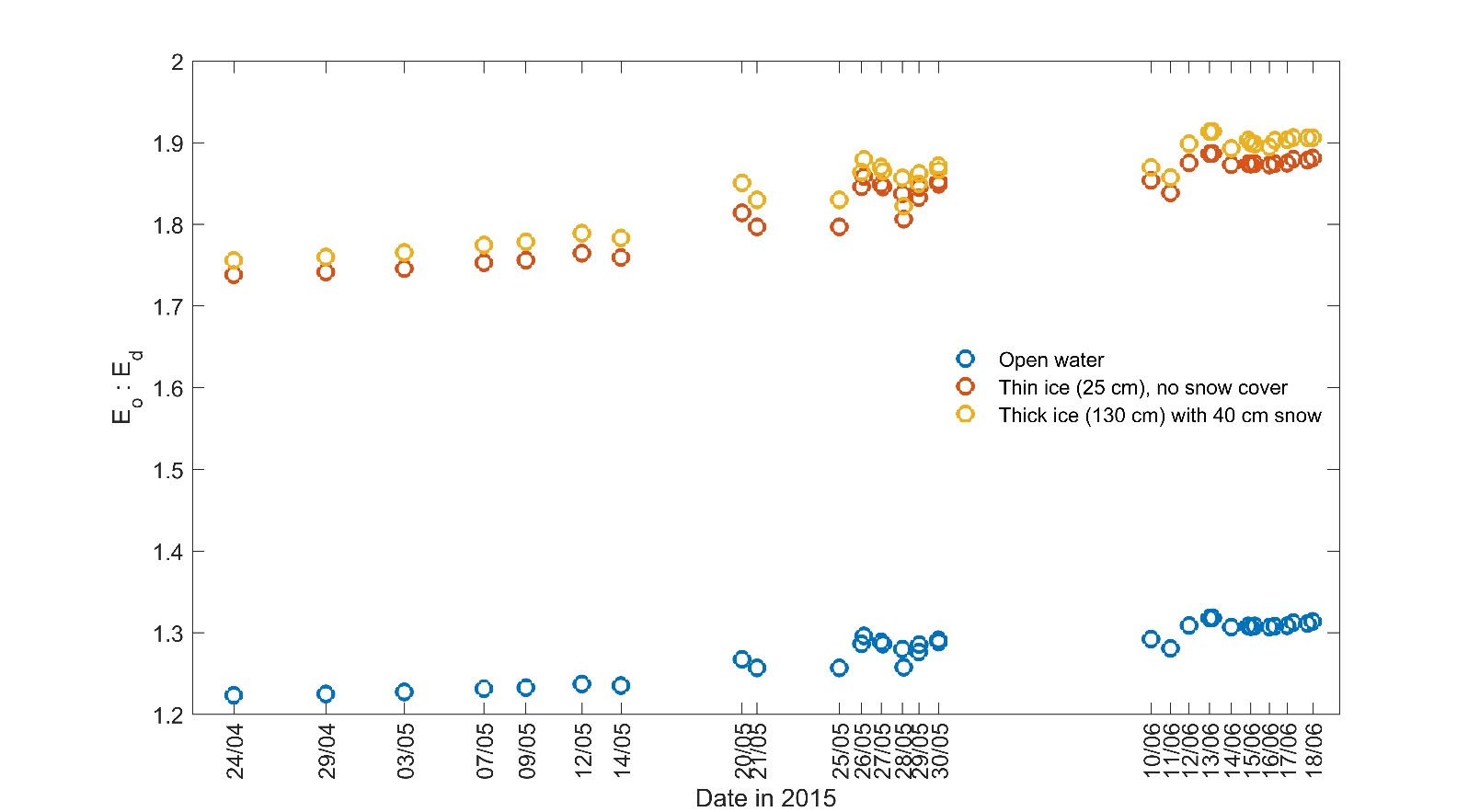


**Figure S6 |** Time series of scalar (E_o_) to downwelling planar (E_d_) irradiance ratio just below the sea ice or sea surface (in case of open water).

**6.2 Primary production model**

The main purpose of the primary production model was to test the growth potential of *Phaeocystis pouchetii* under the aggregate light field (accounting for aerial fractions of thin ice with little snow cover, thicker ice with thick snow cover and open water) encountered during the bloom period. The intention was not to represent a fully realistic model integrating all relevant processes, such as nutrient limitation, grazing, non-grazing mortality or advective processes but simply to test the hypothesis that *P. pouchetii* was able to grow below the snow-covered ice pack interspersed with leads.

A mathematical model solving equation 1 is used to estimate the potential increase in carbon biomass during the bloom period (25 May – 21 June 2015):

$\frac{\Delta B}{\Delta t}=NPP-E$ (1)

Where, *B –* biomass (mg C or mg Chl *a* m^-3^); *NPP* – net primary production; *E* – exudation. Rates are in mg C or mg Chl *a* m^-3^ d^-1^. Conversions between carbon and Chl *a* are carried out assuming a C:Chl *a* ratio of 31.4, based on particulate organic carbon and Chl *a* data obtained between the surface and 25 m depth during the bloom period (not shown). A value corresponding to 33% of the production rate was assumed for *E*, considering that up to one third of *Phaeocystis pouchetii* total photosynthate can be allocated to the colonial matrix^1^.

The model was forced by measured incident photosynthetic active radiation (E_PAR_)^2^ and water temperature^3^, averaged over the upper 40 m. The model uses hourly E_PAR_ radiation, the areal fractions of open water, thin and thick ice (Fig. S7), and ice and snow thicknesses to compute the underwater light field at each time step (1 hour), taking into account water, snow and ice optical properties. Ice and open water fractions (*Fi*) were derived from satellite data (Fig. S4) after interpolation to a daily time step. Thin ice fraction included only ice with a thickness ≤ 25 cm and 2 cm of snow. Snow and ice mean thicknesses were obtained from extensive field surveys. The procedure used to calculate under ice light intensity follows Light et al. (2008)^4^ and it was described in Duarte et al. (2015)^5^. Albedos of sea ice and snow and ice attenuation were used to compute the fraction of incident E_PAR_ that is transmitted to the ocean for each surface type. The Lambert-Beer law was used to calculate the light decay through the snow, ice and water, using different E_PAR_ extinction coefficients. For thick ice with thick snow cover, a published ice extinction coefficient of 0.93 m^-1^ was used^4^ and the snow extinction coefficient adjusted so that calculated (using snow and ice extinction) and observed E_PAR_ transmittances are equal, leading to a snow extinction coefficient of 14.8 m^-1^. For thin ice with thin snow, we made transmittance measurements with snow and with snow removed in a re-frozen lead, the latter gives us the extinction for the thin ice (3.7 m^-1^), and using that value for ice, we can estimate the extinction of the snow layer (37 m^-1^) from the transmittance measurement with snow. All calculations are based on E_PAR_ measurements carried out during the expedition^2^ (not shown).

In the water column, E_PAR_ extinction coefficients (m^-1^) are calculated as a function of observed Chl *a* concentration using the following equation^6^.

$K=0.04+0.0088\bullet Chl+0.054\bullet e^{2/3\bullet\ln(Chl)}$ (2)

*In situ* IOP measurements in the water column and simulations with the radiative transfer model AccuRT (see section 6.1 above) showed that scalar irradiances were significantly higher than downwelling planar irradiance used to force the model. The difference increased with scattering, in direct relation with the increased Chl *a* concentration during the bloom period. In addition, the difference depended on the type of surface (ice/open water). Correction factors were calculated to account for this bias, under the assumption that scalar irradiance is more relevant for primary production calculations than downwelling planar irradiance. These factors were 1.9 below ice and 1.3 below the surface of open water areas (Fig. S6). Simulations were carried out with and without this correction for a more conservative assessment of the growth potential.

The Platt production-light (P-E) function^7,8^ is used for light limitation:

$f(E_{PARz} ) =\left[1-\exp\left(-\frac{\alpha\bullet E_{PARz} }{P_{s}^{B} }\right)\right]\bullet\exp\left(-\frac{\beta\bullet E_{PARz} }{P_{s}^{B} }\right)$ (3)

Where, *E_z_* (μmol photons m^-2^ s^-1^) is E_PAR_ irradiance at depth *z*;* α* (mg C mg Chl *a*^-1^ h^-1^ μmol photons m^-2^ s^-1^) is the initial slope and *β* (mg C mg Chl *a*^-1^ h^-1^ μmol photons m^2^ s^1^) is the negative slope of the P-E curve at high light intensities, reflecting the strength of photoinhibition. Light limitation (equation 3) is integrated as a function of depth, to return vertically averaged *f(E_z_)* values, and weight averaged for the open water and the thin and thick ice fractions (equation 4):

(4)

*Fi* is the fractional area of each of the three surface types (see above).

The depth integration was performed numerically using the Euler method and it implied calculating irradiance at several intermediate depths between *Z_0_* and *Z_1_*, applying equation 3.

Temperature limitation is calculated based on earlier work^9,10^.

(5)

Where *TempAugRate* = 0.0663 ^o^C^-1^ and *T_0_* = 0 ^o^C^-1^.

*NPP* is calculated from the following equation multiplying the maximum photosynthetic rate by light and temperature limitation and biomass standing stock to return the result in aerial units:

(6)

Where, $P_{s}^{B}$is the maximum photosynthetic rate (mg C mg Chl *a*^-1^ h^-1^) and *B* is expressed in mg Chl *a* m^-3^. The parameters of the P-E Platt function were taken from a study conducted in the northern Greenland Sea^1^ and are synthesized in Supplementary Table S4. In the present study it is assumed that they correspond to NPP. The result of equation 6 and exudation are used to estimate the growth potential with equation 1.

Figure 3a shows the time evolution of open water and the ice fractions derived from satellite data (see section 5) and incident E_PAR_ (cf. – Methodology). Simulations carried out with diatoms and with *P. pouchetii* started with identical biomass corresponding to a Chl *a* concentration of 1.3 mg m^-3^, as observed when we first encountered the bloom on 25 May 2015. Diatom biomass increase was negligible (<1 mg m^-3^). However, *P. pouchetii* increased to almost 28 mg m^-3^ over the bloom period. When the scalar to downwelling planar E_PAR_ correction factors were not applied, biomass increase was lower (~ 17 mg m^-3^) (Fig. 3b), but twice as much as maximum observed Chl *a* concentrations during the bloom. NPP remained low (< 10 % of $P_{s}^{B}$) due to the low irradiances.

The model results presented here maybe biased by not accounting for effects of mortality and hydrodynamic biomass transport. However, predicted biomass increase was considerably larger than observed. This suggests that the high photosynthetic efficiency of *P. pouchetii* allows it to grow and develop a bloom of the magnitude observed, even under low light conditions.

**Table S4 |** P-E curve parameter values were averaged from results obtained with algae collected at the surface and at a depth corresponding to 0.1% surface light, in the case of *Phaeocystis pouchetii*, or at depths corresponding to 50 and 1% of surface light, in the case of diatoms^1^.

|  | ***α*** | ***β*** | $P_{s}^{B}$ (mg C mg Chl *a*^-1^ h^-1^) |
| --- | --- | --- | --- |
|  | (mg C mg Chl *a*^-1^ h^-1^ µmol photons m^2^ s^1^) | |
| *P. pouchetii* | 0.09 | 2.6X10^-3^ | 13.2 |
| Diatoms | 0.01 | 0.8X10^-4^ | 1.0 |

**Figure S7 |** The model uses the open water fraction and the areal fractions and thicknesses of two ice types representative of the study region to compute aggregate under-water irradiance at each time step (1 h), taking into account water, snow and ice optical properties. Horizontal and vertical light variability are integrated to calculate primary production.

**References**

1. Cota, G.F., Smith, W.O.Jr. & Mitchell, B. Photosynthesis of *Phaeocystis* in the Greenland Sea. *Limnol. Oceanogr.* **39,** 948-953 (1994).
2. Taskjelle, T., Hudson, S. R., Pavlov, A., & Granskog, M. A. N-ICE2015 surface and under-ice spectral shortwave radiation data (v1.4) [Data set]. Norwegian Polar Institute. doi:10.21334/npolar.2016.9089792e (2016).
3. N-ICE2015 ocean microstructure profiles (MSS90L). Norwegian Polar Institute (Tromsø, Norway): <https://data.npolar.no/dataset/774bf6ab-b27e-51ab-bf8c-eb866cf61be2> (2017).
4. Light, B., Grenfell, T.C. & Perovich, D.K. Transmission and absorption of solar radiation by Arctic sea ice during the melt season. *J. Geophys. Res.* **113,** C03023 (2008).
5. Duarte, P., Assmy, P., Hop, H., Spreen, G., Gerland, S. & Hudson, S.R. The importance of vertical resolution in sea ice algae production models. *J. Marine Syst.* **145,** 69-90 (2015).
6. Parsons, T.R., Takahashi, M. & Hargrave, B. Biological oceanographic processes, 3rd ed. Pergamon Press, Oxford (1984).
7. Platt, T., Gallegos, C.L. & Harrison, W.G. Photoinhibition of photosynthesis in natural assemblages of marine phytoplankton. *J. Mar. Res.* **38,** 687-701 (1980).
8. Platt, T., Harrison, W.G., Irwin, B., Horne, E.P. & Gallegos, C.L. Photosynthesis and photoadaption of marine phytoplankton in the Arctic. *Deep-Sea Res.* **29,** 1159-1170 (1982).
9. Moore, J.K., Doney, S.C., Kleypas, J.A., Glover, D.M. & Fung, I.Y. An intermediate complexity marine ecosystem model for the global domain. *Deep-Sea Res. PT II* **49,** 403-462 (2002).
10. Jin, M., Deal, C. & Wang, J. A coupled ice-ocean ecosystem model for I-D and 3-D applications in the Bering and Chukchi Seas. *Chinese J. Pol. Sci.* **19,** 218- 229 (2008).
11. **Photosynthetic parameters**

**Table S5 |** Photosynthetic parameters of phytoplankton during the bloom period obtained from Phyto-PAM measurements: maximum quantum yield of photosystem II fluorescence (Φ_PSIImax_, in dark-acclimated cells), light saturation parameter (E_k_) in µmol photons m^-2^ s^-1^, maximum light utilization coefficient (α), and max8imum relative electron transfer rate (rETR_max_) derived from Rapid Light Curves. Phytoplankton <60 µm was excluded from the phytoplankton sample taken on 25 May with a multinet (MN) (64 µm mesh size) but *Phaeocystis* colonies were retained. All other samples were taken with Niskin bottles or a hand-held water sampler.

| Date (2015) | Depth (m) | Φ_PSIImax_ | E_k_ | α | rETR_max_ |
| --- | --- | --- | --- | --- | --- |
| 25 May | MN (0-20) | 0.48 | 464 | 0.188 | 87 |
| 1 June | 5 | 0.64 | 584 | 0.259 | 151 |
| 1 June | 15 | 0.66 | 254 | 0.295 | 75 |
| 2 June | 2 | 0.60 | 297 | 0.274 | 73 |
| 2 June | 5 | 0.61 | 377 | 0.241 | 91 |
| 2 June | 15 | 0.60 | 246 | 0.262 | 64 |
| 6 June | 5 | 0.65 | 230 | 0.274 | 63 |
| 11 June | Under ice | 0.52 | 404 | 0.257 | 104 |
| 11 June | 5 | 0.53 | 378 | 0.197 | 74 |
| 11 June | 25 | 0.64 | 137 | 0.287 | 39 |
| 12 June | 15 | 0.59 | 265 | 0.276 | 73 |

1. **Silicic acid**


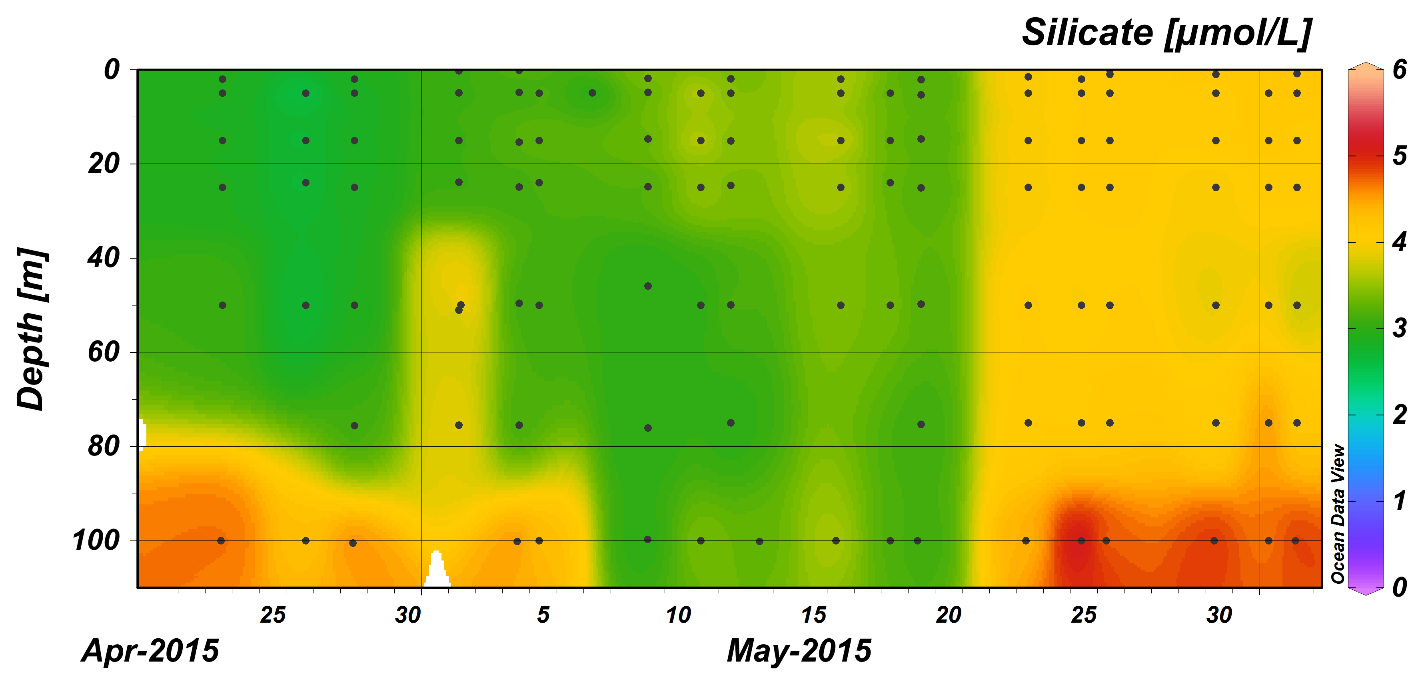


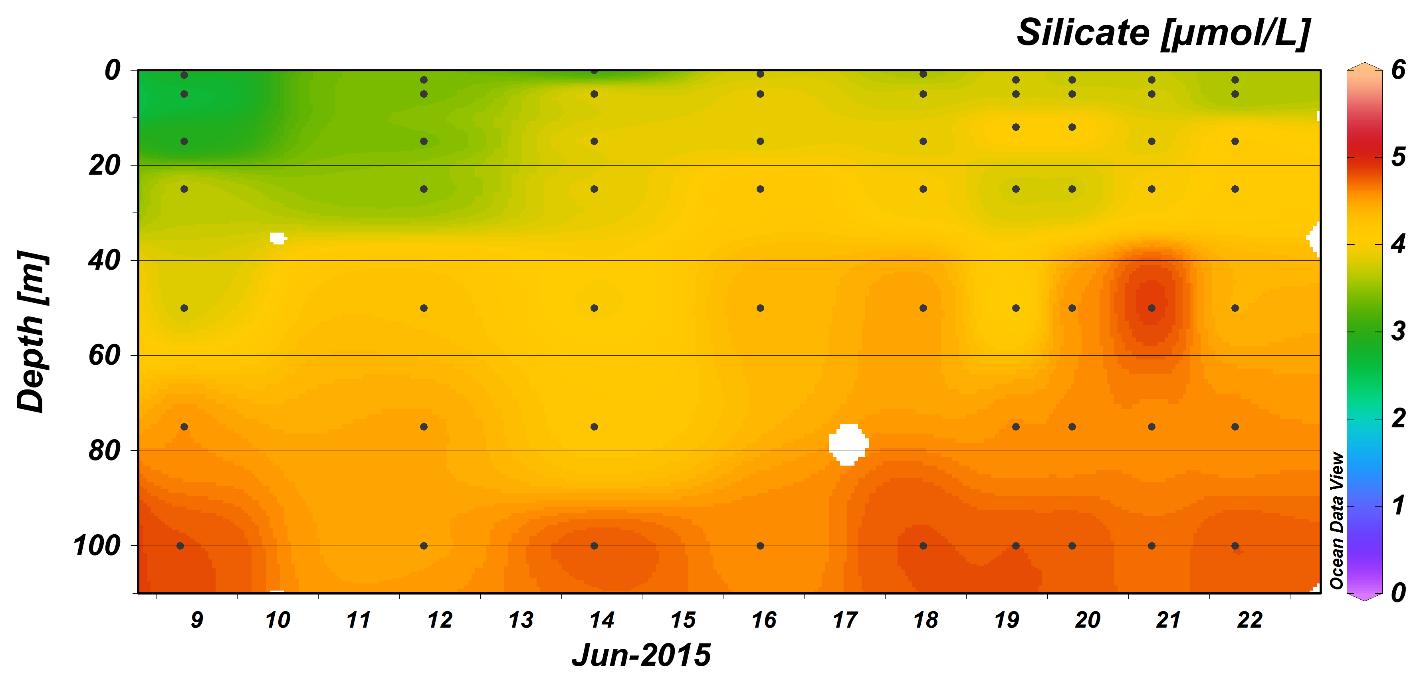


**Figure S8 |** Silicic acid concentrations (in µmol L^-1^) during drift of floe 3 (upper panel) and floe 4 (lower panel) for the upper 100m of the water column. Note that the abrupt increase in silicic acid concentration between 19 and 23 May (upper panel) is due to changes in water mass properties and not silicic acid utilization. In contrast, the reduction in surface silicic acid concentration in early June (lower panel) was due to uptake by diatoms (see elevated diatom biomass on 8 June in Fig. 2a). Figure S8 was generated with Ocean Data View version 4.7.8 ([odv.awi.de](http://odv.awi.de/))^1^.

**References**

1. Schlitzer, R., Ocean Data View. [odv.awi.de](http://odv.awi.de/) (2015).
2. **Modelled ocean circulation and water mass characteristics**

**PSY4 operational model:**

Hindcasts from the global 1/12° real time operational system (PSY4 in the main text) developed at Mercator Ocean for the Copernicus Marine Environment Monitoring Service (CMEMS; http://marine.copernicus.eu/) are used. The system is based on the NEMO (Nucleus for European Modelling of the Ocean^1^) platform and uses a multi-data and multivariate reduced order Kalman filter based on the singular extended evolutive Kalman (SEEK) filter formulation. The model has 50 z-levels with 1 m resolution at the surface, uses the LIM2 thermodynamic-dynamic sea ice model and is driven at the surface by atmospheric analysis and forecasts from the Integrated Forecasting System (IFS) operational at the European Centre for Medium-Range Weather Forecasts (ECMWF). A 3D-VAR temperature and salinity bias correction scheme is included. The assimilated observations are along-track satellite altimetry, sea surface temperature and *in situ* profiles of temperature and salinity^2^. The data assimilation scheme is switched off in ice-covered areas, but is fully operational in the Nordic seas.

**Water mass definitions in the PSY4 operational model:**

Polar Surface Water is defined as water with density <27.8 kg m^-3^.

Atlantic Water is defined as water with density >27.8 kg m^-3^.

**Table S6 |** Modelled (PSY4 operational model) and observed (vessel mounted profiling current meter) mean absolute net velocities in cm s^-1^ during the bloom period for floe 3 (25 May–3 June) and floe 4 (7–18 June) in subsurface waters (20-30 m). The observed velocities error associated with the sensor is ± 0.5 cm s^-1^.

|  | **Velocity components (cm s^-1^)** | **Floe 3** | **Floe 4** | **Bloom period** |
| --- | --- | --- | --- | --- |
| **PSY4 model**  **(no tides)** | U | -0.6 | -1.3 | -1.0 |
|  | V | 0.0 | -0.1 | 0.0 |
| **Observations (including tides)** | U | -2.0 | -2.3 | -2.2 |
|  | V | 0.1 | 0.0 | 0.1 |

**References**

1. Madec, G. & the NEMO team: NEMO ocean engine, Note du Pôle de modélisation 27, Institut Pierre-Simon Laplace (IPSL), France, ISSN 1288–1619 (2008).
2. Lellouche, J.-M. *et al.* Evaluation of global monitoring and forecasting systems at Mercator Océan. *Ocean Sci.* **9,** 57-81 (2013).

Supplementary video caption:

Animation of Norwegian Meteorological Institute ice charts from 16 April to 19 June 2015 demonstrating the variability of the sea ice edge position from before and during the observed under-ice phytoplankton bloom. For this study we define the sea ice edge as the boundary between the open water class (sea ice concentrations lower than 10%) and classes of very open drift ice and above (sea ice concentrations 10% and greater). The software used to create the ice chart animation was Python 2.7.6 (<https://www.python.org/>) and ImageMagick 6.7.7-10 ([http://www.imagemagick.org](http://www.imagemagick.org/)).
