# Supplementary figures and images for: Leads in Arctic pack ice enable early phytoplankton blooms below snow-covered sea ice
Source: Sci Rep. 2017 Jan 19;7:40850. doi: 10.1038/srep40850 (PMC5244362; doi:10.1038/srep40850)

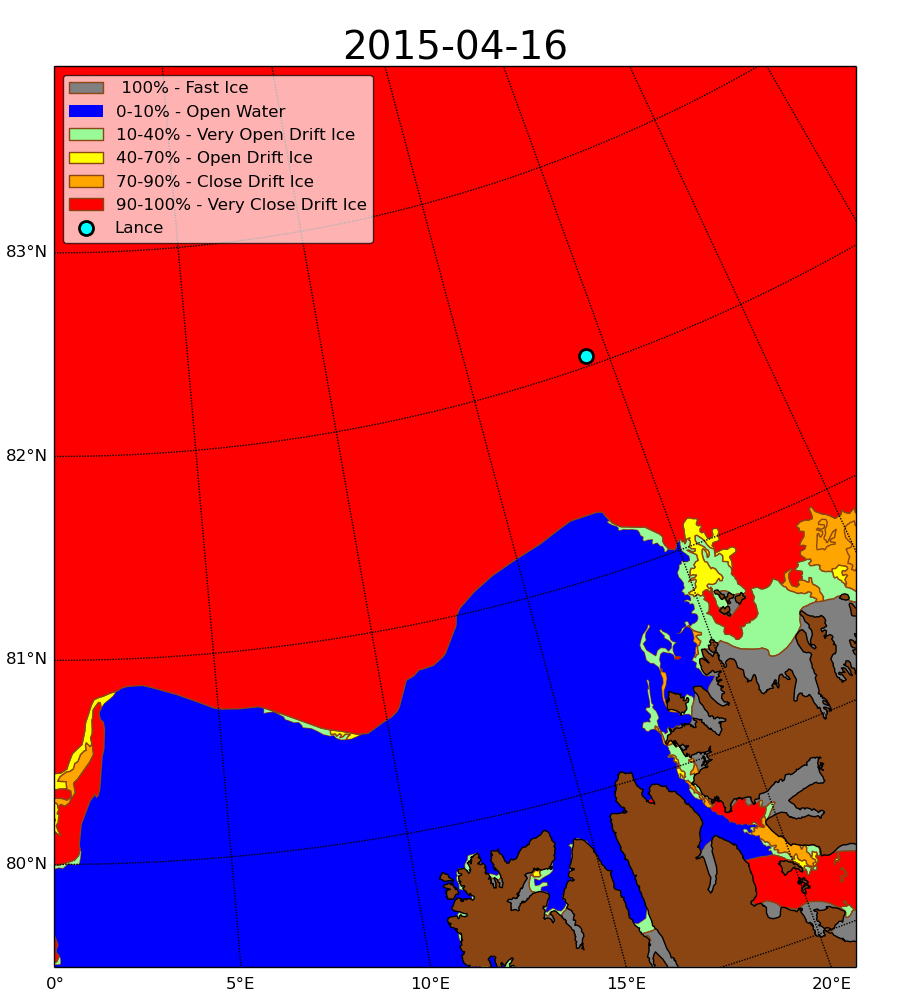

Supplement: Supplementary Video [file srep40850-s2.gif]
